# Temporal Metagenomic and Metabolomic Characterization of Fresh Perennial Ryegrass Degradation by Rumen Bacteria

**DOI:** 10.3389/fmicb.2016.01854

**Published:** 2016-11-18

**Authors:** Olga L. Mayorga, Alison H. Kingston-Smith, Eun J. Kim, Gordon G. Allison, Toby J. Wilkinson, Matthew J. Hegarty, Michael K. Theodorou, Charles J. Newbold, Sharon A. Huws

**Affiliations:** ^1^Institute of Biological, Environmental and Rural Sciences, Aberystwyth UniversityAberystwyth, UK; ^2^Department of Animal Science, Kyungpook National UniversitySangju, Korea; ^3^Department of Animal Production, Welfare and Veterinary Sciences, Harper Adams UniversityNewport, UK

**Keywords:** metagenomics, microbiome, rumen, bacteria, colonization, perennial ryegrass, plant degradation

## Abstract

Understanding the relationship between ingested plant material and the attached microbiome is essential for developing methodologies to improve ruminant nutrient use efficiency. We have previously shown that perennial ryegrass (PRG) rumen bacterial colonization events follow a primary (up to 4 h) and secondary (after 4 h) pattern based on the differences in diversity of the attached bacteria. In this study, we investigated temporal niche specialization of primary and secondary populations of attached rumen microbiota using metagenomic shotgun sequencing as well as monitoring changes in the plant chemistry using mid-infrared spectroscopy (FT-IR). Metagenomic Rapid Annotation using Subsystem Technology (MG-RAST) taxonomical analysis of shotgun metagenomic sequences showed that the genera *Butyrivibrio, Clostridium, Eubacterium, Prevotella*, and *Selenomonas* dominated the attached microbiome irrespective of time. MG-RAST also showed that *Acidaminococcus, Bacillus, Butyrivibrio*, and *Prevotella* rDNA increased in read abundance during secondary colonization, whilst *Blautia* decreased in read abundance. MG-RAST Clusters of Orthologous Groups (COG) functional analysis also showed that the primary function of the attached microbiome was categorized broadly within “metabolism;” predominantly amino acid, carbohydrate, and lipid metabolism and transport. Most sequence read abundances (51.6, 43.8, and 50.0% of COG families pertaining to amino acid, carbohydrate and lipid metabolism, respectively) within these categories were higher in abundance during secondary colonization. Kyoto encyclopedia of genes and genomes (KEGG) pathways analysis confirmed that the PRG-attached microbiota present at 1 and 4 h of rumen incubation possess a similar functional capacity, with only a few pathways being uniquely found in only one incubation time point only. FT-IR data for the plant residues also showed that the main changes in plant chemistry between primary and secondary colonization was due to increased carbohydrate, amino acid, and lipid metabolism. This study confirmed primary and secondary colonization events and supported the hypothesis that functional changes occurred as a consequence of taxonomical changes. Sequences within the carbohydrate metabolism COG families contained only 3.2% of cellulose activities, on average across both incubation times (1 and 4 h), suggesting that degradation of the plant cell walls may be a key rate-limiting factor in ensuring the bioavailability of intra-plant nutrients in a timely manner to the microbes and ultimately the animal. This suggests that a future focus for improving ruminant nutrient use efficiency should be altering the recalcitrant plant cell wall components and/or improving the cellulolytic capacity of the rumen microbiota.

## Introduction

Due to a growing population and increased demand for livestock products by developing countries, current projections estimate that global demand for meat and milk will have doubled by 2050 compared to the start of the twenty-first century (Foresight, 2011). Ruminants supply much of our red meat and nearly all our milk supplies globally. Therefore, there is a real challenge to ensure sustainability and efficiency of ruminant production given that land is also at a premium due to increasing bioenergy crop production. A major hurdle in increasing ruminant productivity is that the conversion of plant to microbial protein is inefficient. As little as 30% of the ingested nitrogen is utilized by ruminants for milk or meat production, and the non-incorporated nitrogen is excreted to the environment as urea or ammonia (MacRae and Ulyatt, 1974; Dewhurst et al., 1996; Kingston-Smith et al., 2008, 2010).

The rumen, via it's complex microbiome is responsible for the breakdown of plant material and the functional capacity of the microbiome defines the amount, quality, and composition of meat and milk produced, whilst also defining the release of nitrogen and greenhouse gases to the environment (Edwards et al., 2008a; Kim et al., 2009; Kingston-Smith et al., 2010; Brown Kav et al., 2012; Huws et al., 2014a). The process of colonizing ingested plant material by the rumen microbiome is rapid (Cheng et al., 1980; Miron et al., 2001; Russell and Rychlik, 2001; Koike et al., 2003a; Edwards et al., 2007, 2008b; Huws et al., 2013, 2014b, 2016), and eventually these populations form mature biofilms encompassed in self-produced polymeric substances (EPS) (Akin, 1976; Cheng et al., 1980, 1981; McAllister et al., 1994; Huws et al., 2013; Leng, 2014). We have previously shown that bacterial diversity attached to fresh perennial ryegrass (PRG) incubated within the rumen is different prior to 4 h and post 4 h of incubation, thus demonstrating that colonization undergoes primary (up to 4 h) and secondary (after 4 h) events, respectively (Huws et al., 2013, 2014b, 2016). Nonetheless, the functionality of the primary and secondary colonizers in terms of plant degradation and availability of nutrients to the host remains unclear.

In this study, we investigated the diversity and function of the attached microbiota using metagenomic based shotgun Illumina sequencing to gain insight into their function and importance in terms of plant degradation and subsequent nutrient availability to the microbes and ultimately the ruminant. Temporal changes in plant chemistry were also monitored using Fourier transform infrared spectroscopy (FT-IR) to confirm that microbial gene abundances were related to substrate changes within the plant itself. Understanding temporal bacterial-driven plant degradation and factors controlling these events will promote the development of novel strategies to increase ruminant production in order to meet the increasing demand for meat and milk.

## Materials and methods

### Growth and preparation of plant material

PRG (*Lolium perenne* cv. AberDart) was grown from seed in plastic seed trays (length 38 cm × width 24 cm × depth 5 cm) filled with compost (Levingtons general purpose). The trays were housed in a greenhouse under natural irradiance with additional illumination provided during the winter months (minimun 8 h photoperiod). A temperature of 22/19°C day/night was maintained and plants were watered twice a week. Plants were harvested after 6 weeks and cut 3 cm above soil level, before washing in cold distilled water and cutting with scissors into 1 cm sections. Sub-samples of plant material were freeze-dried and stored at −20°C for metagenomic sequencing, plant dry matter (DM) and Fourier transform mid-infrared spectroscopy (FT-IR) analysis (0 h samples).

### *In vitro* incubations

Cut PRG (7.5 g) was added to Duran bottles (250 mL) together with anaerobic incubation buffer (135 mL pre-warmed to 39°C; Van Soest, 1967) and rumen fluid inoculum (15 mL, strained through two layers of muslin and held under CO_2_ at 39°C; rumen fluid was taken from three cannulated cows grazed mainly on fresh forage and pooled before inoculation). Rumen fluid was obtained from cannulated cows under the authority of Licenses under the UK Animal Scientific Procedures Act, 1986. Bottles were incubated in a horizontally rotating rack at 100 rpm and 39°C (Incubator-shaker, LA Engineering, UK). Bottle contents were harvested at 0.25, 0.5, 1, 2, 4, 8, and 24 h. At each time interval bottle contents were harvested by vacuum filtration through filter paper (11 μm^2^ pore size; ®QL100, Fisher Scientific, Leicestershire, UK). Retained plant material was washed with phosphate buffered saline (PBS; 50 mL) to remove loosely attached bacteria, before attached bacteria were removed by incubation overnight in gluteraldehyde (3 % v/v in PBS) at 4°C (Azeredo et al., 1999). The remaining plant material from which colonizing bacteria had been removed was retrieved by squeezing contents of overnight gluteraldehyde incubations through one layer of muslin. Plant residues within the muslin were then freeze-dried and weighed to allow calculation of percentage plant degradation. Absence of remaining attached bacteria was also checked using Quantitative PCR (QPCR), as described below, to validate the method of detachment of attached microbes. The plant material was subsequently finely ground with the aid of a reciprocal shaking system in the presence of liquid nitrogen (particle size <0.5 mm) for Fourier transform infrared spectroscopy (FT-IR) analyses. The suspension of previously attached bacteria (supernatant retrieved post squeezing of overnight gluteraldehyde incubations) was centrifuged (10,000 x g, 10 min), before the pellet was freeze-dried for subsequent QPCR and metagenomic sequencing. The experiment was repeated on three separate occasions (*n* = 3) within the same week.

### DNA extraction, QPCR and metagenomic sequencing

DNA extraction and total bacterial 16S rDNA QPCR were completed as described by Huws et al. (2013) and Huws et al. (2016) using the primers 5′-GTG STG CAY GGY TGT CGT CA-3′ (Forward) and 5′-GAG GAA GGTGKG GAY GAC GT-3′ (Reverse) (Maeda et al., 2003). The presence of primary and secondary bacterial colonizing events were initially corroborated using denaturing gradient gel electrophoresis (DGGE) as described by Huws et al. (2013) before taxonomy and function of the primary and secondary attached microbiome was further investigated by sequencing. Essentially, 1 (primary colonizers) and 4 h (secondary colonizers) DNA samples from the attached microbiome were sequenced using Illumina HiSeq DNA was normalized to 0.2 ng/μL and 1 ng used to create metagenomic libraries using the Nextera® XT DNA kit (Invitrogen, San Diego, USA) following manufacturer guidelines. Sample libraries were sequenced at 2 × 151 bp using an Illumina HiSeq 2500 rapid run, following standard manufacturer's instructions at the IBERS Aberystwyth Translational Genomics Facility. Reads of 126 bases were merged and first trimmed to remove the Nextera library adapters then trimmed at the 3′-end when a sliding window over four bases fell below an average Phred Q score of 30. Sequences are deposited and publically available within Metagenomic Rapid Annotation using Subsystem Technology (MG-RAST) [Identification numbers 4653312.3 (1 h), 4653313.3 (1 h), 4653314.3 (1 h), 4653315.3 (4 h), 4653316.3 (4 h), and 4653317.3 (4 h)].

### Metagenomic sequence analysis

Sequencing files containing the merged, quality trimmed reads, were uploaded to MG-RAST (Meyer et al., 2008) as FASTQ files. The MG-RAST best hit organism abundance function was employed against the RDP comparison pipeline, using constraints of 97% sequence similarity and a maximum *e*-value of 1 × 10^−5^ and minimum sequence alignment of 15, to assign taxonomy to sequences. The MG-RAST functional abundance hierarchical abundance function was employed against the COG database to assign function, based on a maximum *e*-value of 1 × 10^−5^, minimum identity cut-off of 60% and minimum sequence alignment of 15. Normalized taxonomic and functional abundance data were exported as excel files for statistical analysis. Heatmap and bar chart visualization of gene function data was completed within MG-RAST. Eukaryotic rRNA sequences were removed from the analysis (these made up on average 51.6% of the reads obtained and were mainly 18S rDNA from PRG); the COG database does not annotate eukaryote sequences allowing an analysis of taxonomy and function of the rumen prokaryotic microbiota only (Li et al., 2016).

### Fourier transform infrared spectroscopy (FT-IR)

Mid infrared spectra reflecting plant chemical composition were obtained at each time point by attenuated total reflectance (ATR) FT-IR analysis using a Bruker Equinox 55 spectrometer (Bruker Optics Ltd., Coventry, UK) equipped with a deuterated tryglycine sulfate detector and a Golden Gate ATR accessory (Specac Ltd., Orpington, UK). Spectra were acquired over the range 4000 to 500 cm^−1^ as a mean of 32 scans and at a spectral resolution of 4 cm^−1^ using OPUS software (version 4.2, Bruker Optics Ltd., Coventry, UK).

### Statistical analysis

For plant degradation, QPCR data, taxonomy, and COG gene abundance data, significant differences between groups was determined using one-way analysis of variance (ANOVA) followed by Duncan's multiple range tests to detect significant differences between groups where appropriate (Duncan, 1955) using the GenStat program (Tenth Edition, VSN International Ltd., Hemel Hemstead, UK; Payne et al., 2007). Taxonomy and function based PCoA plots, function based-bar charts, heatmaps, and KEGG pathways were generated in MG-RAST. *T*-test were conducted within MG-RAST using level 2 COG based bar chart data in order to compare differences in gene abundances for the broad classifications within primary and secondary colonization events. FT-IR spectra were converted to text files using OPUS and imported to Matlab for statistical and chemometricanalysis using Matlab (version 6.5.1) and the Matlab Statistics Toolbox (version 4, The Mathworks, Cambridge, UK). Spectra were analyzed for underlying structure correlating with incubation time by principal component analysis (PCA) (Martens and Naes, 1989; Mariey et al., 2001; Sheng et al., 2006) and significant differences between spectra from different time points were detected using multivariate one-way analysis of variance model (MANOVA). In all instances the threshold of statistical significance was set at *P* < 0.05.

## Results

### Plant dry matter disappearance and attached bacterial 16S rDNA abundance

Residual plant digestibility data (with microbes removed) showed that by 4 h 13.6% of the plant material had been degraded with this increasing to 76.2% by 24 h (Table 1). Post incubation of PRG under rumen-like *in vitro* conditions, rumen bacteria attached quickly to the plant material, and bacterial 16S rDNA abundance increased rapidly and continued to rise for the duration of the experiment; by 4 h attached bacterial 16S rDNA concentration had increased by 1.9X, and by 24 h by 2.6X compared with 0 h concentrations (Table 1).

**Table 1:** **Plant dry matter degradation and concentration of attached bacterial 16S rDNA following incubation of fresh perennial ryegrass in the presence of rumen fluid**.

	**Incubation time (h)**									
	**0.0**	**0.25**	**0.5**	**1**	**2**	**4**	**8**	**24**	**SED**	***P***
Dry matter degradation (%) (g DM lost from 100 g of initial DM)	0.0^a^	1.6^a^	2.3^a^	4.2^a^	7.9^ab^	13.6^b^	35.2^c^	76.2^d^	3.52	<0.001
Solid-associated bacteria [SAB] (Log_10_ bacterial DNA concentration [ng g^−1^ RDM])	1.8^a^	2.3^ab^	2.3^ab^	2.4^ab^	2.7^b^	3.4^c^	4.4^dc^	4.7^d^	0.2	<0.001

One-way ANOVA was conducted on effect of incubation time. The results are the mean values of triplicate data sets. RDM, remaining dry matter; SED, standard errors of differences of means. Values within the same row with different superscripts were significantly different (P < 0.05).

### Sequencing data

Post quality control of sequences we obtained on average 0.9 GB/sample, with a mean sequence length of 163 bp (Supplementary Table 1). Rarefaction curves showed that all samples approached a plateau, suggesting reasonable sequence coverage (Supplementary Figure 2).

### Taxonomy of the primary and secondary attached microbiota

**Figure 1:**
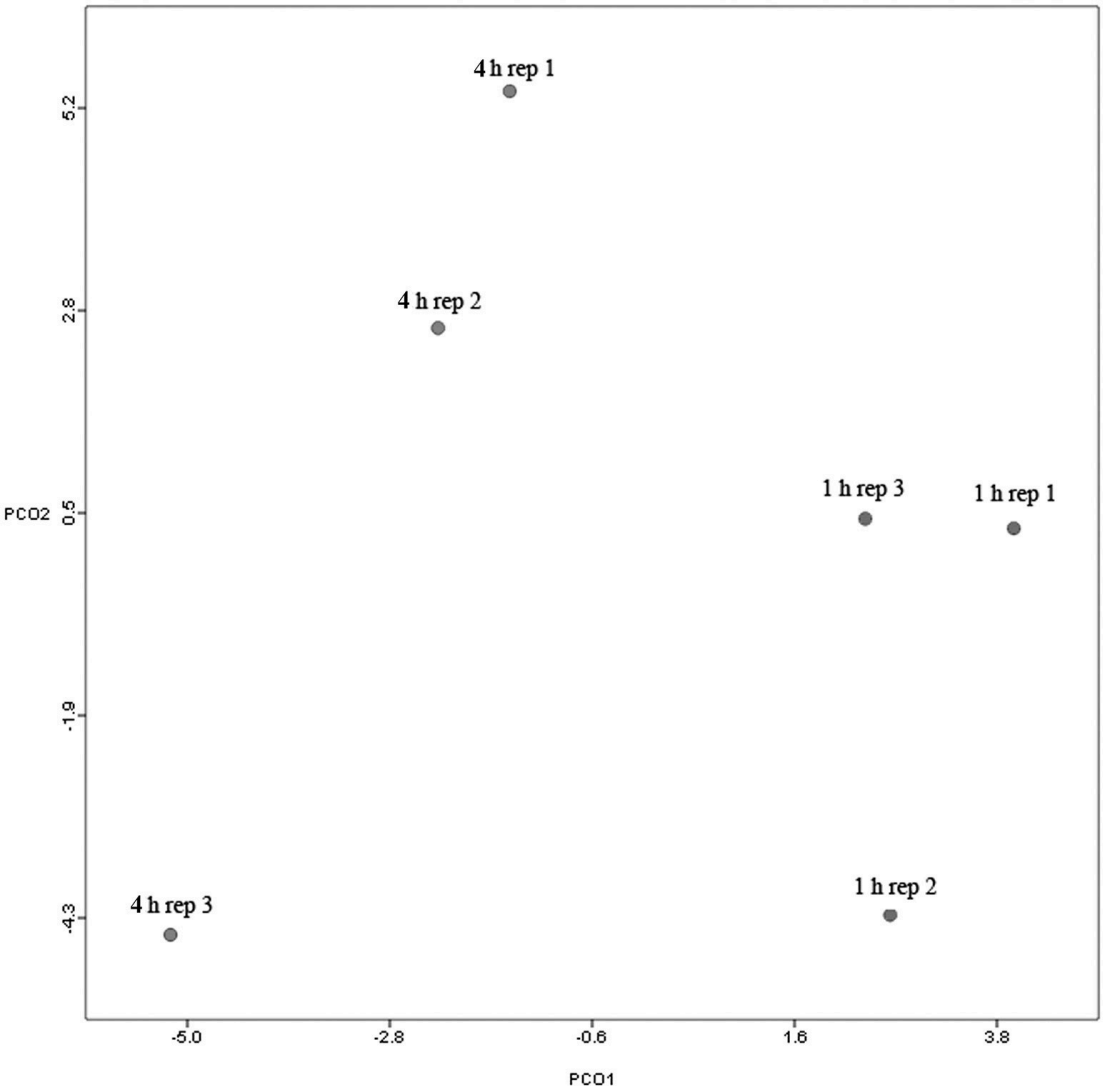
**Principal coordinates analysis (PCoA) analysis of taxonomical classifications of attached bacteria present after 1 and 4 h of rumen incubation**. Rep: Replicate.

Principal coordinate axis (PCoA) plots showed that bacterial diversity differed between 1 and 4 h of colonization (Figure 1). DGGE was also undertaken on samples for all time points and confirmed the presence of primary (up to 4 h) and secondary (post 4 h) bacterial colonization events (Supplementary Figure 1). Phyla level taxonomy showed that the most abundant attached phyla were Firmicutes (66–72% of total read abundances) and Bacteroidetes (15–20% of total read abundances) (Table 2). No significant changes (*P* > 0.05) in read abundances were evident at a phyla level between primary (1 h) and secondary (4 h) colonization events (Table 2). On an order level, Clostridiales (46–51% of total read abundances), Selemonadales (18% of total read abundances), and Bacteroidales (14–15% of total read abundances) were the most abundant, with the remaining orders representing <3% on average of the total read abundances (Table 3). No significant changes (*P* > 0.05) in read abundances were evident at an order level between primary (1 h) and secondary (4 h) colonization events (Table 3). Family level taxonomy showed that the most abundant classified families were *Lachnospiraceae* (25–33% of total read abundances), *Veillonellaceae* (18% of total read abundances), *Prevotellaceae* (11–12% of total read abundances), *Eubacteriaceae* (5–10% of total read abundances), *Clostridiaceae* (5–7% of total read abundances), and *Ruminococcaceae* (3–4% of total read abundances), with the remaining families representing <3% on average of total read abundances (Table 4). Significant (*P* < 0.05) increases in *Bacillaceae, Lachnospiraceae, Porphyromonadaceae*, and *Prevotellaceae* were seen during secondary colonization events compared with their rDNA gene abundances present during primary colonization (Table 4). On a genera level, *Butyrivibrio* (20–23% of total read abundances), *Selenomonas* (17–18%), *Prevotella* (10–13%), *Eubacterium* (5–10%), *Pseudobutyrivibrio* (4–6%), and *Ruminococcus* (3%) were the most abundant, with the remaining genera representing <3% on average of total read abundances (Table 5). Significant (*P* < 0.05) increases in *Acidaminococcus, Bacillus, Blautia, Butyrivibrio*, and *Prevotella*, were also seen during secondary colonization events compared with their rDNA gene abundances present during primary colonization (Table 5).

**Table 2:** **Comparison of the primary (1 h) and secondary (4 h) colonizing bacterial phyla attached to perennial ryegrass**.

**Phylum**	**Time (h)**			
	**1**	**4**	**SED**	***P***
*Actinobacteria*	71	116	34.4	NS
*Bacteroidetes*	453	571	48.5	NS
*Fibrobacteres*	20.7	10.7	9.57	NS
*Firmicutes*	1536	2748	676.3	NS
*Proteobacteria*	37	55	26.2	NS
*Spirochaetes*	36	53	32.7	NS
*Tenericutes*	32.3	41.7	18.84	NS
*Verrucomicrobia*	2	1	1	NS
Unclassified	132	233	64.4	NS

Data shown are for normalized reads. NS: Not Significant.

**Table 3:** **Comparison of the primary (1 h) and secondary (4 h) colonizing bacterial orders attached to perennial ryegrass**.

**Order**	**Time (h)**			
	**1**	**4**	**SED**	***P***
*Acholeplasmatales*	19.5	18.7	14.86	NS
*Aeromondales*	7.3	8.7	8.88	NS
*Anaeroplasmatales*	5.3	5.7	2.62	NS
*Bacilliales*	42	140	87.5	NS
*Bacteroidales*	375	517	61.2	NS
*Clostridiales*	1038	1883	379.4	NS
*Coriobacteridae*	34.7	55.3	11.71	NS
*Cytophagales*	18	7.7	3.3	NS
*Erysipelotrichales*	4.3	15.3	11.48	NS
*Fibrobacterales*	20.7	10.7	9.57	NS
*Flavobacteriales*	20.3	10.3	10.42	NS
*Lactobacillales*	27.7	31	9.48	NS
*Mycoplasmatales*	16.5	7.7	4.97	NS
*Rickettsiales*	4.5	2.33	1.95	NS
*Selenomondales*	411	655	241.6	NS
*Sphingobacteriales*	12.5	5.3	5.4	NS
*Spirochaetales*	36	53	32.7	NS
*Thermoanaerobacterales*	9.3	23.7	5.56	NS
Unclassified	147	248	64.6	NS

Data shown are for normalized reads. NS: Not Significant.

**Table 4:** **Comparison of the primary (1 h) and secondary (4 h) colonizing bacterial families attached to perennial ryegrass**.

**Family**	**Time (h)**			
	**1**	**4**	**SED**	***P***
*Acidaminococcaceae*	11	21	7.16	NS
*Anaeroplasmataceae*	5.3	5.7	2.62	NS
*Bacillaceae*	18.7	51.7	8.1	0.015
*Bacteroidaceae*	73	93	29.1	NS
*Clostridiaceae*	105	235	50.2	NS
*Coriobacteriaceae*	34.7	50.7	14.6	NS
*Erysipelotrichaceae*	4.3	15.3	11.5	NS
*Eubacteriaceae*	215	169	166.6	NS
*Fibrobacteraceae*	20.7	10.7	9.6	NS
*Flavobacteriaceae*	24	9.7	13.9	NS
*Lachnospiraceae*	556	1166	151.9	0.016
*Lactobacillaceae*	8	10	5.1	NS
*Mycoplasmataceae*	11.7	7.7	6.5	NS
*Paenibacillaceae*	8	82	76	NS
*Peptococcaceae*	11.7	38.7	17.6	NS
*Peptostreptococcaceae*	19	20.3	10.6	NS
*Porphyromonadaceae*	16.7	47.7	5.4	0.005
*Prevotellaceae*	273	378	31.1	0.028
*Ruminococcaceae*	80	112	38.2	NS
*Sphingobacteriaceae*	8	5.3	3.5	NS
*Spirochaetaceae*	36	52	32.3	NS
*Streptococcaceae*	18	17.5	9.6	NS
*Streptomycetaceae*	12.7	19.5	10	NS
*Succinivibrionaceae*	10	8.3	9.9	NS
*Thermoanaerobacteraceae*	8.3	13.7	5.2	NS
*Veillonellaceae*	400	638	238.7	NS
Unclassified	230	287	114	NS

Data shown are for normalized reads. NS: Not Significant.

**Table 5:** **Comparison of the primary (1 h) and secondary (4 h) colonizing bacterial genera attached to perennial ryegrass**.

**Genus**	**Time (h)**			
	**1**	**4**	**SED**	***P***
*Acidaminococcus*	4	10.7	2.03	0.03
*Anaeroplasma*	5.3	5.7	2.62	NS
*Anaplasma*	3	3.3	2.24	NS
*Atopobium*	12	17	7.96	NS
*Bacillus*	17	49.7	7.22	0.011
*Bacteroides*	73	93	29.1	NS
*Blautia*	16	60.7	12.21	0.022
*Butyrivibrio*	429	800	107.6	0.026
*Caldanaerobius*	5.7	12.7	4.38	NS
*Cellulosilyticum*	2.7	5.7	3.77	NS
*Clostridium*	102	225	47.5	NS
*Collinsella*	5	2	2.08	NS
*Eubacterium*	214	168	166.4	NS
*Faecalibacterium*	14.3	21.7	11.6	NS
*Fibrobacter*	20.7	10.7	9.6	NS
*Flavobacterium*	4.5	5	2.48	NS
*Gordonibacter*	8	10.3	3.23	NS
*Halothermothrix*	2.7	2.3	0.75	NS
*Hespellia*	4.3	5.7	1.8	NS
*Lactobacillus*	8	10	5.1	NS
*Odoribacter*	1.3	5.7	2.75	NS
*Paenibacillus*	7	82	76	NS
*Parabacteroides*	5	16.7	6.84	NS
*Phascolarctbacterium*	7	7	3.32	NS
*Porphyromonas*	9.7	18.3	11.7	NS
*Prevotella*	270	358	25.8	0.027
*Pseudobutyrivibrio*	74	211	72	NS
*Roseburia*	11.7	23.7	6.5	NS
*Ruminococcus*	63	89	26.3	NS
*Selenomonas*	386	616	231.8	NS
*Slackia*	4.67	7	1.86	NS
*Streptococcus*	13.7	15.7	7.97	NS
*Streptomyces*	17.5	14	10.44	NS
*Tissierella*	9	9.7	9.48	NS
*Treponema*	31	40	31.5	NS
Unclassified	211	401	83.5	NS

Data shown are for normalized reads. NS: Not Significant.

### Functionality of the primary and secondary attached microbiota

**Figure 2:**
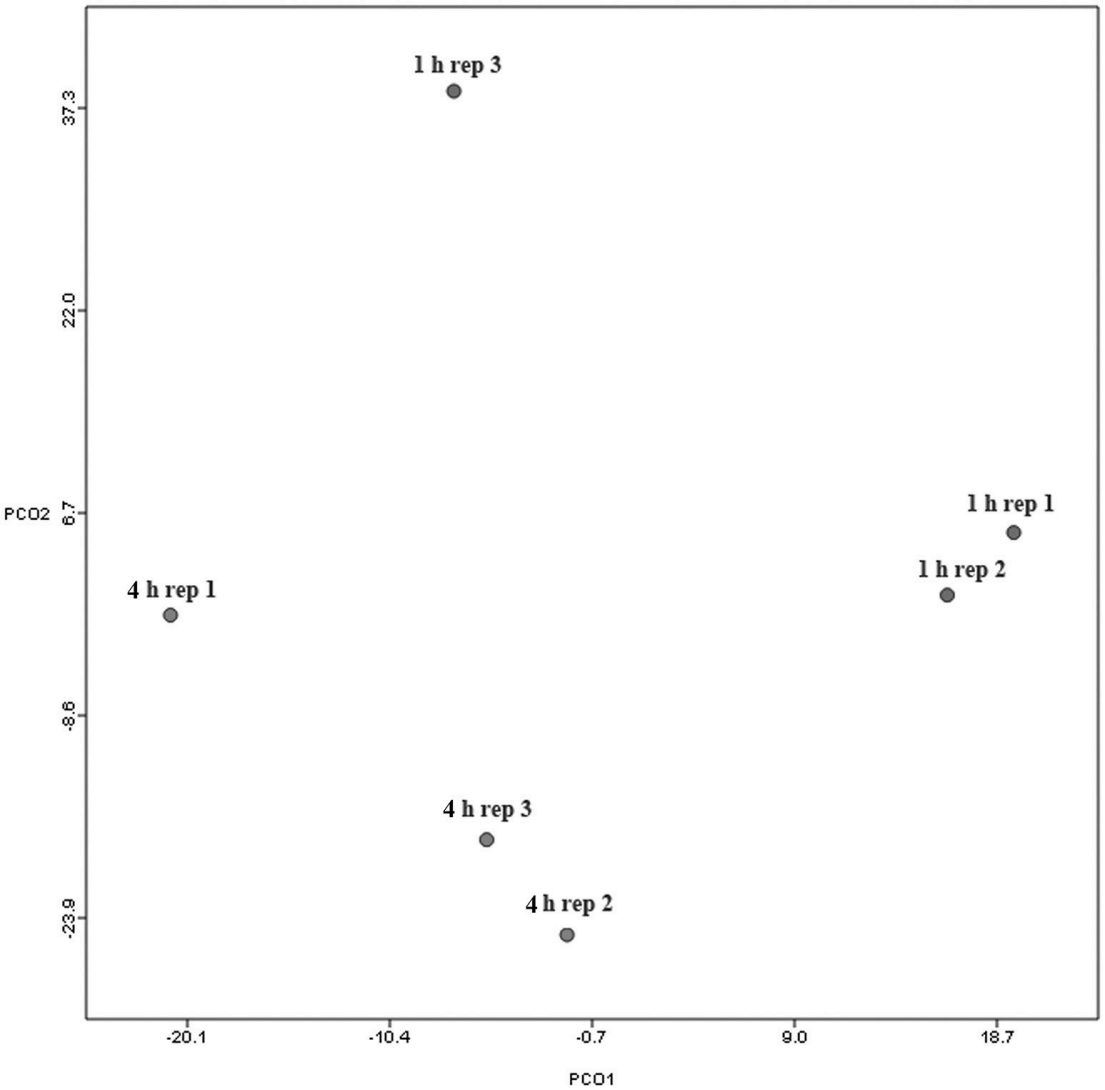
**Principal coordinates analysis (PCoA) analysis of functional classifications of attached bacteria present after 1 and 4 h of rumen incubation**. Rep: Replicate.

**Figure 3:**
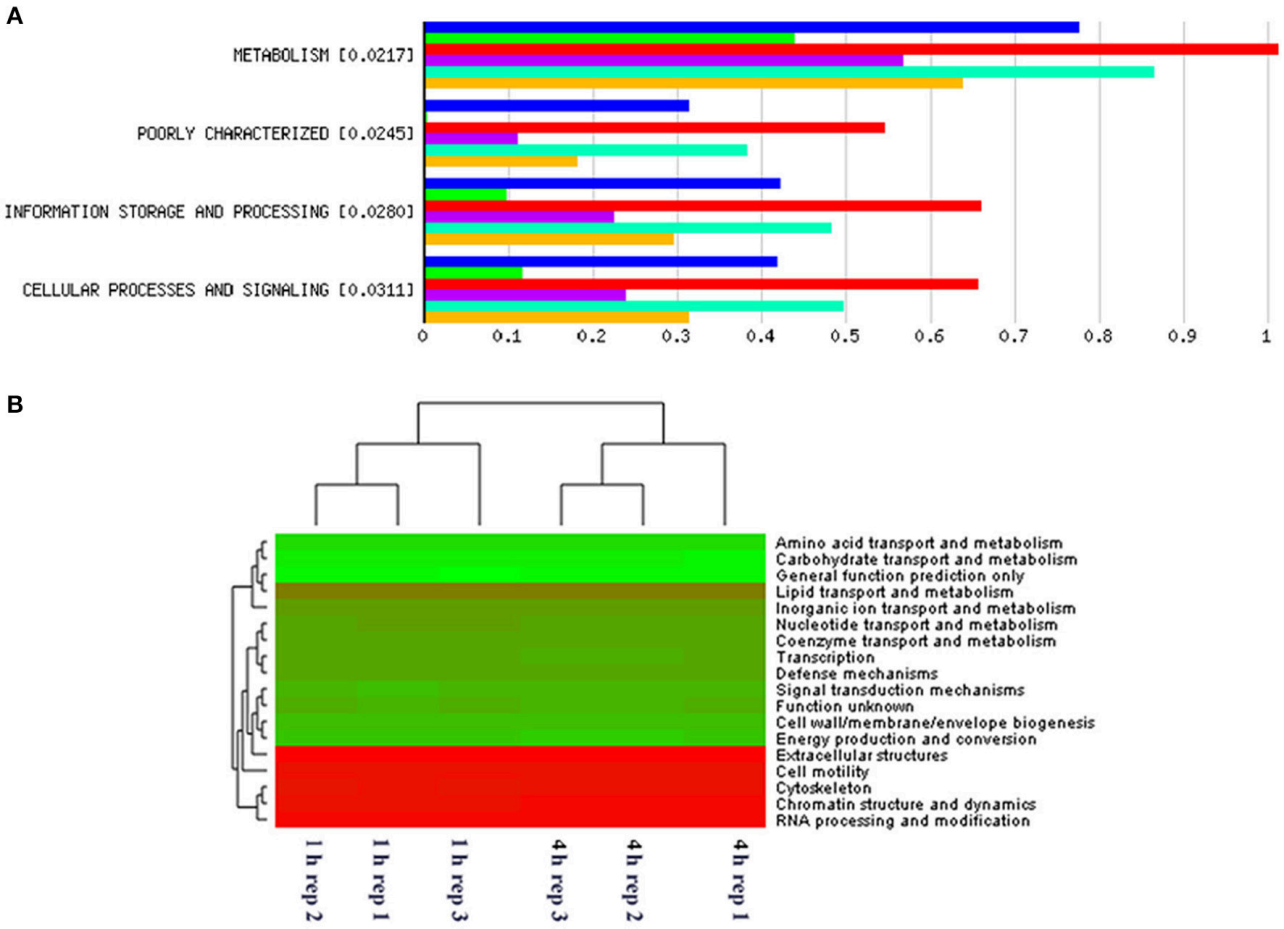
**MG-RAST generated bar chart showing differences in gene abundances within primary and secondary perennial ryegrass bacterial attachment events following rumen like incubation [Blue, red, and turquoise bars (triplicate data) show gene abundances for bacteria attached to perennial ryegrass following 4 h of rumen like incubation (secondary colonization) and green, purple, and yellow bars (triplicate data) show gene abundances for bacteria attached to perennial ryegrass following 1 h of rumen like incubation (primary colonization)] with the numbers in brackets denoting significance level when primary and secondary colonizing bacteria gene abundances were compared using *t*-tests (A)**. MG-RAST generated heatmap of COG level 2 absolute gene abundances within primary (1 h) and secondary (4 h) perennial ryegrass attached bacteria following rumen like incubation (the more intense the green color, the more abundant those COG families are with red denoting low abundance COG families) **(B)**.

**Figure 4:**
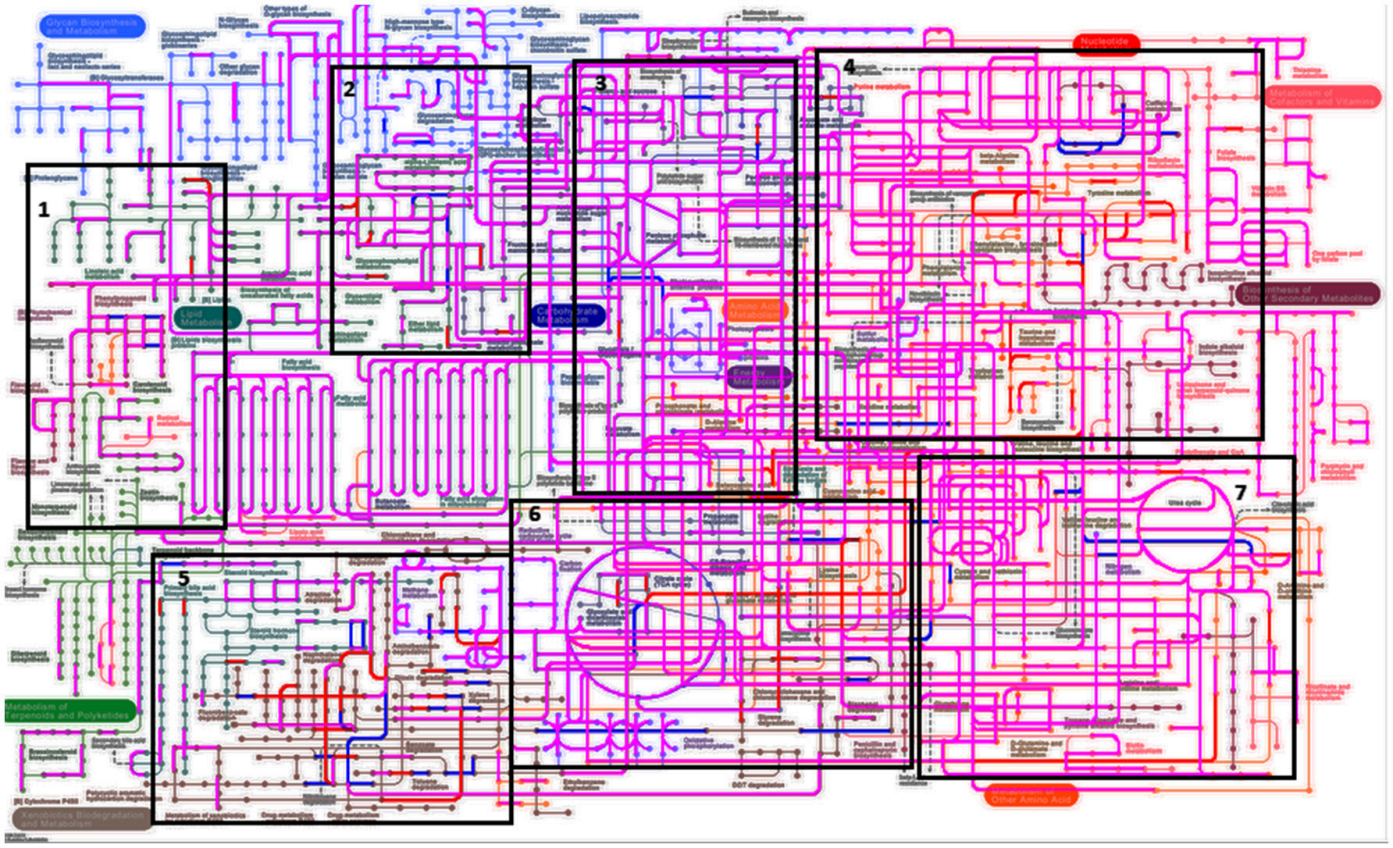
**Kyto encyclopedia of genes and genomes (KEGG) pathways exhibited by perennial ryegrass attached rumen bacteria following 1 and 4 h of rumen incubation**. Blue lines show pathways present by plant attached bacteria following 1 h of rumen incubation. Red lines show pathways present by plant attached bacteria following 4 h of rumen incubation. Pink/Purple lines show pathways present by plant attached bacteria at both 1 and 4 h of rumen incubation. Boxes 1–7 have been denoted in order to formulate Table 9 listing pathways in blue and red in order to note unique pathways present at one incubation time only.

When assessing bacterial functional gene abundance at 1 and 4 h post rumen incubation, PCoA plots showed that bacterial function differed between 1 and 4 h of colonization (Figure 2). Bar charts generated within MG-RAST based on hierarchical functional categories showed that genes attributed broadly within metabolism, information, storage, and processing and cellular processes and signaling were significantly (*P* > 0.05) more abundant within secondary colonizing bacteria than they were within the population of primary colonizers (Figure 3A). The COG level 2 heatmap corroborated the difference in functionality seen within the primary and secondary colonizing bacteria (Figure 3B). The heatmap also illustrated that the primary functions of the attached microbiota were amino acid transport and metabolism, carbohydrate transport and metabolism, general function, lipid transport, and metabolism (Figure 3B). Further prospecting of amino acid transport and metabolism COG families showed that 51.6% of the COG families showed significant (*P* < 0.05) increases in abundance from primary (1 h) to secondary (4 h) colonization events (Table 6). No COG families decreased in significantly in their abundance from primary (1 h) to secondary (4 h) colonization events (Table 6). Further prospecting of carbohydrate transport and metabolism COG families showed that 43.8% of the COG families showed significant (*P* < 0.05) increases in abundance, whilst only 0.01% significant (*P* < 0.05) decreased in abundance between primary (1 h) to secondary (4 h) colonization events (Table 7). Only 3.2% of total reads pertained to cellulases (Table 7). Further prospecting of lipid transport and metabolism COG families showed that 50.0% of the COG families showed significant (*P* > 0.05) increases from primary (1 h) to secondary (4 h) colonization events (Table 8). No COG families decreased significantly in abundance from primary (1 h) to secondary (4 h) colonization events (Table 8). COG families with a low abundance (<1) were omitted from Tables 6–8. KEGG pathway analysis (Figure 4) corroborated that most functional pathways were present within PRG attached bacteria at both 1 and 4 h post rumen incubation with only a few being uniquely found in the attached bacteria at 1 or 4 h of incubation only (Table 9).

**Table 6:** **Amino acid transport and metabolism clusters of orthologous genes (COG) categories showing significant read abundance differences within the primary (1 h) and secondary (4 h) bacteria attached to perennial ryegrass**.

**COG number**	**Function**	**Average COG normalized read abundance**			
		**1 h**	**4 h**	**SED**	***P***
2	Acetylglutamate semialdehyde dehydrogenase	38	94.3	16.2	0.026
6	Xaa-Pro aminopeptidase	87	148	21.9	0.049
10	Arginase/agmatinase/formimionoglutamate hydrolase, arginase family	11.7	14	4.48	NS
14	Gamma-glutamyl phosphate reductase	77.3	163	18.39	0.01
19	Diaminopimelate decarboxylase	115	221	32	0.029
31	Cysteine synthase	93	166	23.6	0.038
40	ATP phosphoribosyltransferase	39.3	64.7	6.45	0.017
65	3-Isopropylmalate dehydratase large subunit	68	133	21.2	0.036
69	Glutamate synthase domain 2	206	384	59.4	0.04
70	Glutamate synthase domain 3	182	345	47.9	0.027
75	Serine-pyruvate aminotransferase/archaeal aspartate aminotransferase	35	62.7	9.74	0.047
76	Glutamate decarboxylase and related PLP-dependent proteins	21	28.3	7.64	NS
77	Prephenate dehydratase	34.7	61	11.54	NS
78	Ornithine carbamoyltransferase	76	158	29.3	0.05
79	Histidinol-phosphate/aromatic aminotransferase and cobyric acid decarboxylase	7.3	13	0.8	0.003
82	Chorismate synthase	45.7	92.3	17.52	NS
83	Homoserine kinase	51	88.3	4.4	0.001
106	Phosphoribosylformimino-5-aminoimidazole carboxamide ribonucleotide (ProFAR)	41	70.3	11.32	NS
107	Isomeraseimidazoleglycerol-phosphate synthase	74	160	23.5	0.022
111	Phosphoglycerate dehydrogenase and related dehydrogenases	107	206	33.6	0.05
112	Glycine/Serine hydroxymethyltransferase	43	77.7	7.6	0.01
118	Glutamine amidotransferase	159	308	36	0.014
119	Isopropylmalate/Homocitrate/Citramalate synthases	70.3	130.3	9.4	0.003
128	Tryptophan synthase beta chain	49	89	17.87	NS
131	Imidazoleglycerol-phosphate dehydratase	58.7	97.7	16.7	NS
133	Tryptophan synthase beta chain	23	40.7	4.7	0.019
134	Phosphoribosylanthranilate isomerase	27.7	49.7	8.6	NS
135	Phosphoribosylanthranilate isomerase	59.7	121.3	17.6	0.025
136	Aspartate-semialdehyde dehydrogenase	62.3	131	14.4	0.009
137	Argininosuccinate synthase	27.3	46.3	6.8	0.049
139	Phosphoribosyl-AMP cyclohydrolase	31	54	8.64	NS
140	Phosphoribosyl-ATP pyrophosphohydrolase	69.7	131.7	18.8	0.03
141	Histidinol dehydrogenase	86	173	27.4	0.034
159	Tryptophan synthase alpha chain	31.7	49.3	6.92	NS
165	Argininosuccinate lyase	52.7	86.7	8.3	0.015
169	Shikimate 5-dehydrogenase	27.3	51	10.48	NS
174	Glutamine synthetase	108	218	41.9	NS
241	Histidinol phosphatase and related phosphatases	36.3	55.3	8.69	NS
253	Diaminopimelate epimerase	12	25	3.3	0.016
260	Leucyl aminopeptidase	8.3	9.3	4.03	NS
263	Glutamate 5-kinase	45.7	81.7	15.28	NS
287	Prephenate dehydrogenase	23	48.3	6.9	0.021
289	Dihydrodipicolinate reductase	145	263	27.8	0.013
308	Aminopeptidase N	42	45.7	11.22	NS
334	Glutamate dehydrogenase/Leucine dehydrogenase	25.7	54.3	8	0.023
337	3-Dehydroquinate synthetase	15	37.3	5.7	0.017
339	Zn-dependent oligopeptidases	26.3	52.3	8.5	0.038
345	Pyrroline-5-carboxylate reductase	25.3	43	5.1	0.026
346	Lactoylglutathione lyase and related lyases	51	85	20.8	NS
347	Nitrogen regulatory protein PII	147	283	22.9	0.004
367	Asparagine synthase (glutamine-hydrolyzing)	85	185	29.9	0.028
403	Glycine cleavage system protein P (pyridoxal-binding), N-terminal domain	18	34.7	7.9	NS
404	Glycine cleavage system T protein (aminomethyltransferase)	13	23.7	6.57	NS
405	Gamma-glutamyltransferase	12.7	18.3	6.55	NS
410	ABC-type branched-chain amino acid transport systems, ATPase component	56.7	105.7	13.7	0.023
421	Spermidine synthase	350	651	84	0.023
436	Aspartate/Tyrosine/Aromatic aminotransferase	73.7	126.7	19	0.05
440	Acetolactate synthase, small (regulatory) subunit	26	46.3	13.82	NS
460	Homoserine dehydrogenase	115	187	24.2	0.04
462	Phosphoribosylpyrophosphate synthetase	38.7	80.7	13.82	NS
473	Isocitrate/isopropylmalate dehydrogenase	69	126	25	NS
498	Threonine synthase	97	166	40.1	NS
506	Proline dehydrogenase	4.3	12.3	2.98	NS
509	Glycine cleavage system H protein (lipoate-binding)	5.35	8	1.563	NS
520	Selenocysteine lyase	83.3	159.3	19.9	0.019
527	Aspartokinases	51.3	85	11.5	0.043
547	Anthranilate phosphoribosyltransferase	29.7	40.7	9.91	NS
548	Acetylglutamate kinase	15.3	37	5.9	0.021
549	Carbamate kinase	17.7	35	5.5	0.034
559	Branched-chain amino acid ABC-type transport system, permease components	37.3	80.7	19.15	NS
560	Phosphoserine phosphatase	17.7	36.7	6.3	0.044
620	Methionine synthase II (cobalamin-independent)	26.3	64.7	9.4	0.015
624	Acetylornithine deacetylase/Succinyl-diaminopimelate desuccinylase and related deacylases	57	109	21.4	NS
626	Cystathionine beta-lyases/cystathionine gamma-synthases	70	118	27.4	NS
646	Methionine synthase I (cobalamin-dependent), methyltransferase domain	127	206	36	NS
665	Glycine/D-amino acid oxidases (deaminating)	7	8.3	2.19	NS
683	ABC-type branched-chain amino acid transport systems, periplasmic component	26.3	56.7	8	0.019
685	5,10-Methylenetetrahydrofolate reductase	46.3	77.7	10.8	0.043
686	Alanine dehydrogenase	4.7	12.7	2.8	0.044
687	Spermidine/Putrescine-binding periplasmic protein	15.7	38.3	9.07	NS
703	Shikimate kinase	13	18	2.31	NS
710	3-Dehydroquinate dehydratase	57	110.3	19.3	0.051
722	3-Deoxy-D-arabino-heptulosonate 7-phosphate (DAHP) synthase	44.7	94.7	12.6	0.017
747	ABC-type dipeptide transport system, periplasmic component	120.3	236	17.7	0.003
757	3-Dehydroquinate dehydratase II	32	49.7	6.98	NS
765	ABC-type amino acid transport system, permease component	49.3	90.3	9.5	0.013
786	Na+/glutamate symporter	7.3	13.3	4.11	NS
814	Amino acid permeases	13	14	5.32	NS
1003	Glycine cleavage system protein P (pyridoxal-binding), C-terminal domain	29.3	40.3	6.72	NS
1027	Aspartate ammonia-lyase	18.7	30	7.49	NS
1045	Serine acetyltransferase	77	189	29.4	0.019
1104	Cysteine sulfinate desulfinase/Cysteine desulfurase and related enzymes	12	22.7	2.96	0.023
1113	Gamma-aminobutyrate permease and related permeases	24.7	31.7	11.54	NS
1114	Branched-chain amino acid permeases	210	396	58.6	0.034
1115	Na+/Alanine symporter	139	276	57.8	NS
1125	ABC-type proline/glycine betaine transport systems, ATPase components	4.3	6.7	3.35	NS
1126	ABC-type polar amino acid transport system, ATPase component	62	173	24.1	0.01
1164	Oligoendopeptidase F	18.7	33	5.1	0.049
1166	Arginine decarboxylase (spermidine biosynthesis)	54.3	108.3	18.2	0.041
1168	Bifunctional PLP-dependent enzyme with beta-cystathionase and maltose regulon repressor activities	18.3	38.3	6.9	0.044
1171	Threonine dehydratase	34.3	76.3	17.09	NS
1174	ABC-type proline/glycine betaine transport systems, permease component	2	4.67	1.86	NS
1176	ABC-type spermidine/putrescine transport system, permease component I	15.7	41.7	9.84	NS
1177	ABC-type spermidine/putrescine transport system, permease component II	7.7	16	2.3	0.021
1280	Putative threonine efflux protein	4.3	6.7	2.49	NS
1296	Predicted branched-chain amino acid permease (azaleucine resistance)	9.3	49	8.7	0.027
1305	Transglutaminase-like enzymes, putative cysteine proteases	68.7	134.3	9.7	0.003
1362	Aspartyl aminopeptidase	35	71.7	10.9	0.028
1364	N-acetylglutamate synthase (N-acetylornithine aminotransferase)	10.3	24.3	3.8	0.021
1410	Methionine synthase I, cobalamin-binding domain	143	225	39	NS
1446	Asparaginase	7	7.7	2.26	NS
1465	Predicted alternative 3-dehydroquinate synthase	4	6	1.29	NS
1505	Serine proteases of the peptidase family S9A	7	6	2.77	NS
1506	Dipeptidyl aminopeptidases/acylaminoacyl-peptidases	110	165	22.5	NS
1509	Lysine 2,3-aminomutase	28.3	49.3	7.5	0.048
1605	Chorismate mutase	12	25	7.3	NS
1703	Putative periplasmic protein kinase ArgK and related GTPases of G3E family	41	51.3	6.86	NS
1748	Saccharopine dehydrogenase and related proteins	63	124	22.7	0.054
1760	L-serine deaminase	40	79	15.58	NS
1770	Protease II	20	15	6.76	NS
1775	Benzoyl-CoA reductase/2-hydroxyglutaryl-CoA dehydratase subunit, BcrC/BadD/HgdB	7.7	18.3	6.37	NS
1897	Homoserine trans-succinylase	32	91	17.1	0.026
1921	Selenocysteine synthase [seryl-tRNASer selenium transferase]	4	10.7	3.23	NS
1932	Phosphoserine aminotransferase	50.3	92.3	15.3	0.081
1982	Arginine/lysine/ornithine decarboxylases	37.7	63.7	18.43	NS
1984	Allophanate hydrolase subunit 2	4	9.7	2.11	NS
2008	Threonine aldolase	3.7	9.1	1.9	0.052
2021	Homoserine acetyltransferase	10.7	14	2.79	NS
2040	Homocysteine/selenocysteine methylase (S-methylmethionine-dependent)	6.67	8.33	1.491	NS
2049	Allophanate hydrolase subunit 1	11	22.7	2.9	0.016
2066	Glutaminase	103	216	33.9	0.029
2171	Tetrahydrodipicolinate N-succinyltransferase	6.3	10.3	3.35	NS
2195	Di- and tripeptidases	6.3	18.7	2.6	0.009
2235	Arginine deiminase	12	21	3.1	0.042
2303	Choline dehydrogenase and related flavoproteins	11.7	11.3	3.4	NS
2309	Leucyl aminopeptidase (aminopeptidase T)	24	50	11.28	NS
2317	Zn-dependent carboxypeptidase	48	89.7	12.3	0.028
2355	Zn-dependent dipeptidase, microsomal dipeptidase homolog	16	23.7	3.07	NS
2423	Predicted ornithine cyclodeaminase, mu-crystallin homolog	11.7	22.3	6.57	NS
2502	Asparagine synthetase A	54.3	105.7	18.9	0.053
2515	1-Aminocyclopropane-1-carboxylate deaminase	7	8.3	24	NS
2610	H+/Gluconate symporter and related permeases	40.3	68.7	5.4	0.006
2755	Lysophospholipase L1 and related esterases	175	299	39.1	0.034
2873	O-acetylhomoserine sulfhydrylase	6.7	27.7	2.9	0.002
2939	Carboxypeptidase C (cathepsin A)	34	36.3	8.97	NS
2957	Peptidylarginine deiminase and related enzymes	25.3	42	6.51	NS
2986	Histidine ammonia-lyase	7.7	22.7	3.1	0.009
2987	Urocanate hydratase	3.7	11.7	2.4	0.027
3033	Tryptophanase	13	27	6.57	NS
3048	D-serine dehydratase	3.7	11.7	2.36	0.027
3104	Dipeptide/tripeptide permease	87	117	38.4	NS
3283	Transcriptional regulator of aromatic amino acids metabolism	30	64.7	9.9	0.025
3404	Methenyl tetrahydrofolate cyclohydrolase	3	9	1.6	0.021
3579	Aminopeptidase C	15.3	37	2.3	<0.001
3705	ATP phosphoribosyltransferase involved in histidine biosynthesis	8	16.7	6.54	NS
3962	Acetolactate synthase	12.7	27	6.57	NS
4303	Ethanolamine ammonia-lyase, large subunit	5.3	7.7	3.35	NS
4598	ABC-type histidine transport system, ATPase component	3.33	4.67	1.97	NS
4820	Ethanolamine utilization protein, possible chaperonin	4	9	2.16	NS
4917	Ethanolamine utilization protein	7.3	20.3	8.01	NS
4992	Ornithine/acetylornithine aminotransferase	66	134	27.3	NS

NS: Not Significant.

**Table 7:** **Carbohydrate metabolism clusters of orthologous genes (COG) categories showing significant read abundance differences within the primary (1 h) and secondary (4 h) bacteria attached to perennial ryegrass**.

**COG number**	**Function**	**Average COG normalized read abundance**			
		**1 h**	**4 h**	**SED**	***P***
21	Transketolase	60	92.7	15.47	NS
33	Phosphoglucomutase	8.33	9.33	1.599	NS
36	Pentose-5-phosphate-3-epimerase	63	123	24.4	NS
57	Glyceraldehyde-3-phosphate dehydrogenase/erythrose-4-phosphate dehydrogenase	43.7	71.7	6.52	0.013
58	Glucan phosphorylase	205	363	51.7	0.038
120	Ribose 5-phosphate isomerase	3.7	10.7	4.19	NS
126	3-Phosphoglycerate kinase	89.7	164.7	15	0.008
129	Dihydroxyacid dehydratase/phosphogluconate dehydratase	69	125	22.4	NS
148	Enolase	38.3	91.7	14.38	0.021
149	Triosephosphate isomerase	43	83.7	10.14	0.016
153	Galactokinase	45	96.7	10.41	0.008
158	Fructose-1,6-bisphosphatase	5.7	3.7	2.36	NS
166	Glucose-6-phosphate isomerase	60.3	101.7	11.67	0.024
176	Transaldolase	18.3	36	6.61	NS
191	Fructose/Tagatose bisphosphate aldolase	65	123	19.55	0.041
205	6-Phosphofructokinase	108	195	29.8	0.044
235	Ribulose-5-phosphate 4-epimerase and related epimerases and aldolases	84	165	26	0.037
246	Mannitol-1-phosphate/altronate dehydrogenases	91	180	24.8	0.023
269	3-Hexulose-6-phosphate synthase and related proteins	4	12.3	4.52	NS
279	Phosphoheptose isomerase	11.7	23.7	6.94	NS
296	1,4-Alpha-glucan branching enzyme	150	286	50.7	0.054
297	Glycogen synthase	67	146	31.6	0.068
362	6-Phosphogluconate dehydrogenase	22.7	37.3	5.68	0.061
363	6-Phosphogluconolactonase/Glucosamine-6-phosphate isomerase/deaminase	73	141	24	0.047
364	Glucose-6-phosphate 1-dehydrogenase	9.3	7.7	3.2	NS
366	Glycosidases	262	569	88.2	0.025
380	Trehalose-6-phosphate synthase	24.7	16.3	4.53	NS
383	Alpha-mannosidase	26.3	47.7	7.02	0.038
395	ABC-type sugar transport system, permease component	141	337	48.5	0.016
406	Fructose-2,6-bisphosphatase	51.7	43.7	15.94	NS
451	Nucleoside-diphosphate-sugar epimerases	429	764	100.9	0.029
469	Pyruvate kinase	82	136	30.4	NS
524	Sugar kinases, ribokinase family	128	225	32.5	0.04
574	Phosphoenolpyruvate synthase/pyruvate phosphate dikinase	194	400	41.4	0.008
580	Glycerol uptake facilitator and related permeases (Major Intrinsic Protein Family)	38	70.3	14.23	0.085
588	Phosphoglycerate mutase 1	16.3	30.7	7.99	NS
647	Predicted sugar phosphatases of the HAD superfamily	8	15	2.77	NS
662	Mannose-6-phosphate isomerase	21.3	36	8.09	NS
696	Phosphoglyceromutase	57.3	122.7	15.07	0.012
698	Ribose 5-phosphate isomerase RpiB	82	145	29.8	NS
702	Predicted nucleoside-diphosphate-sugar epimerases	23.3	17.7	9.39	NS
726	Predicted xylanase/chitin deacetylase	46	82	22	NS
738	Fucose permease	81.7	137.3	14.09	0.017
800	2-keto-3-deoxy-6-phosphogluconate aldolase	30.7	48.3	7.8	NS
1015	Phosphopentomutase	36.7	75.7	11.18	0.025
1080	Phosphoenolpyruvate-protein kinase (PTS system EI component in bacteria)	31.7	59.3	15.36	NS
1082	Sugar phosphate isomerases/epimerases	24	55	12.06	NS
1086	Predicted nucleoside-diphosphate sugar epimerases	239	425	62.7	0.041
1105	Fructose-1-phosphate kinase and related fructose-6-phosphate kinase (PfkB)	178	329	47.5	0.033
1109	Phosphomannomutase	12	39	7.46	0.022
1129	ABC-type sugar transport system, ATPase component	184	420	77.6	0.038
1172	Ribose/xylose/arabinose/galactoside ABC-type transport systems, permease components	44	108	27.4	NS
1175	ABC-type sugar transport systems, permease components	177	388	66.7	0.034
1263	Phosphotransferase system IIC components, glucose/maltose/N-acetylglucosamine-specific	186	390	103.1	NS
1264	Phosphotransferase system IIB components	175	360	96.9	NS
1299	Phosphotransferase system, fructose-specific IIC component	107	287	30.2	0.004
1312	D-mannonate dehydratase	39	62.7	8.51	0.05
1349	Transcriptional regulators of sugar metabolism	19	37.7	9.06	NS
1363^*^	Cellulase M and related proteins	39.7	68.3	10.09	0.047
1440	Phosphotransferase system cellobiose-specific component IIB	3	7	1.633	NS
1445	Phosphotransferase system fructose-specific component IIB	9.3	38.3	7.67	0.019
1449	Alpha-amylase/alpha-mannosidase	14.7	22.3	2.98	NS
1455	Phosphotransferase system cellobiose-specific component IIC	26	59	20.4	NS
1472	Beta-glucosidase-related glycosidases	473	901	103.9	0.015
1482	Phosphomannose isomerase	42	90.7	1.6	0.008
1486	Alpha-galactosidases/6-phospho-beta-glucosidases, family 4 of glycosyl hydrolases	28	68.3	16.12	NS
1501	Alpha-glucosidases, family 31 of glycosyl hydrolases	22	45	53.7	0.013
1523	Type II secretory pathway, pullulanase PulA and related glycosidases	133	226	33.9	0.052
1548	Predicted transcriptional regulator/sugar kinase	3.33	6	1.563	NS
1554	Trehalose and maltose hydrolases (possible phosphorylases)	9	14.7	2.79	NS
1593	TRAP-type C4-dicarboxylate transport system, large permease component	50	116	21.8	0.039
1621	Beta-fructosidases (levanase/invertase)	79	167	31.5	0.049
1626	Neutral trehalase	5	6.7	2.47	NS
1638	TRAP-type C4-dicarboxylate transport system, periplasmic component	28	57.3	11.88	NS
1640	4-Alpha-glucanotransferase	123	199	20.7	0.022
1803	Methylglyoxal synthase	6.7	13	4.74	NS
1820	N-acetylglucosamine-6-phosphate deacetylase	14.3	28.7	7.23	NS
1830	DhnA-type fructose-1,6-bisphosphate aldolase and related enzymes	4	11	1.16	0.004
1869	ABC-type ribose transport system, auxiliary component	2	3.33	1.563	NS
1877	Trehalose-6-phosphatase	30	23	7.79	NS
1925	Phosphotransferase system, HPr-related proteins	7	9.7	3.28	NS
1974	Beta-galactosidase	76.3	113	19.69	NS
1904	Glucuronate isomerase	45.7	104.7	12.39	0.009
1925	Phosphotransferase system, HPr-related proteins	7	9.7	3.28	NS
1929	Glycerate kinase	32	54.3	8.61	NS
1940	Transcriptional regulator/sugar kinase	66	155	31.8	0.048
2074	2-Phosphoglycerate kinase	41.7	79.7	12.5	0.038
2115	Xylose isomerase	21.7	37	9.46	NS
2133	Glucose/sorbosone dehydrogenases	4.7	12.3	7.91	NS
2160	L-arabinose isomerase	76	132	26.9	NS
2182	Maltose-binding periplasmic proteins/domains	4.67	8.33	1.886	NS
2190	Phosphotransferase system IIA components	77	187	45.7	NS
2211	Na+/Melibiose symporter and related transporters	77	131	27.7	NS
2213	Phosphotransferase system, mannitol-specific IIBC component	29.7	55.3	17.8	NS
2271	Sugar phosphate permease	9.3	6	2.11	NS
2273	Galactose mutarotase and related enzymes	6.3	11	3.97	NS
2376	Sugar phosphate permease	59	97	26.2	NS
2379^*^	Beta-glucanase/Beta-glucan synthetase	10.33	16.33	1.97	0.038
2407	L-fucose isomerase and related proteins	81	137	22.9	NS
2513	PEP phosphonomutase and related enzymes	60.7	97.7	16.11	NS
2706	3-Carboxymuconate cyclase	1	27	24.5	NS
2723	Beta-glucosidase/6-phospho-beta-glucosidase/beta-galactosidase	195	418	97.2	NS
2730	Putative glycerate kinase	78	159.3	15.18	0.006
2731	Beta-galactosidase, beta subunit	7	2.7	2.6	NS
2814	Arabinose efflux permease	5.3	11.7	5.28	NS
2893	Phosphotransferase system, mannose/fructose-specific component IIA	18.3	35.7	13.98	NS
2942	N-acyl-D-glucosamine 2-epimerase	36.7	70	14.2	NS
3001	Fructosamine-3-kinase	19.3	22.7	3.4	NS
3010	Putative N-acetylmannosamine-6-phosphate epimerase	6.7	11.7	2.36	NS
3090^*^	Endoglucanase	9.7	20.3	3.4	0.035
3250	TRAP-type C4-dicarboxylate transport system, small permease component	524	875	90.4	0.018
3265	Gluconate kinase	3.3	9	2.67	NS
3345	Beta-galactosidase/beta-glucuronidase	109	207	33	0.041
3386	Gluconolactonase	8	8.33	0.667	NS
3405	Alpha-galactosidase	24	44	5.63	0.024
3408^*^	Endoglucanase Y	20.7	37.3	5.34	0.036
3414	Phosphotransferase system, galactitol-specific IIB component	2	4.33	1.667	NS
3444	Phosphotransferase system, mannose/fructose/N-acetylgalactosamine-specific component IIB	25	55	22.8	NS
3459	Glycogen debranching enzyme	145	289	37.3	0.018
3534	Cellobiose phosphorylase	147	273	33.2	0.019
3537	Alpha-L-arabinofuranosidase	48.3	98	2.91	<0.001
3588	Fructose-1,6-bisphosphate aldolase	4.7	8.3	2.49	NS
3623	Putative L-xylulose-5-phosphate 3-epimerase	11	15	5.16	NS
3635	Putative alpha-1,2-mannosidase	21	48	7.37	0.022
3661	Predicted phosphoglycerate mutase, AP superfamily	58	115.7	14.11	0.015
3664^*^	Beta-xylosidase	198	363	72.1	NS
3669	Alpha-glucuronidase	73.7	147	18.7	0.017
3693	Alpha-L-fucosidase	68	132	21.1	NS
3715	Phosphotransferase system, mannose/fructose/N-acetylgalactosamine-specific component IIC	36	63	20.6	0.038
3716	Phosphotransferase system, mannose/fructose/N-acetylgalactosamine-specific component IID	44	91	26.1	NS
3717^*^	Beta-1,4-xylanase	41.7	69	8.07	0.028
3730	Phosphotransferase system sorbitol-specific component IIC	6.3	13	3.13	NS
3732	Phosphotransferase system sorbitol-specific component IIBC	12.3	27	12.78	NS
3775	5-keto 4-deoxyuronate isomerase	14.67	23	1.67	0.007
3833	Phosphotransferase system, galactitol-specific IIC component	24	58.7	11.57	0.04
3839	ABC-type maltose transport systems, permease component	127	281	32.5	0.009
3866	Pectate lyase	24	30	5.29	NS
3867	ABC-type sugar transport systems, ATPase components	39.3	82.7	13.12	0.03
3925	N-terminal domain of the phosphotransferase system fructose-specific component IIB	2	1	0.577	NS
3934	Endo-beta-mannanase	8.67	10.67	1.7	NS
3957	Arabinogalactan endo-1,4-beta-galactosidase	33	67.7	10.51	0.03
3958	Phosphoketolase	41.3	86	9.48	0.009
3959	Transketolase, C-terminal subunit	40.3	88.7	15.26	0.034
4124	Beta-mannanase	17.3	28	4.81	NS
4154	Fucose dissimilation pathway protein FucU	2.7	8	2.33	NS
4209	Transketolase, N-terminal subunit	56.3	134	11.94	0.003
4211	ABC-type polysaccharide transport system, permease component	9.3	20	3.67	0.044
4213	ABC-type glucose/galactose transport system, permease component	66	144.3	19.57	0.016
4214	ABC-type xylose transport system, periplasmic component	28.7	60.7	10.61	0.039
4284	UDP-glucose pyrophosphorylase	14.3	20	2.91	NS
4354	ABC-type xylose transport system, permease component	17.3	7.7	2.81	0.026
4409	Predicted bile acid beta-glucosidase	2.67	7.7	1.49	0.028
4468	Galactose-1-phosphate uridyltransferase	28	58	16.45	NS
4573	Predicted tagatose 6-phosphate kinase	2.33	3.67	1.106	NS
4632	Exopolysaccharide biosynthesis protein related to N-acetylglucosamine-1-phosphodiester alpha-N-acetylglucosaminidase	12.7	24	7.31	NS
4668	Mannitol/fructose-specific phosphotransferase system, IIA domain	16	27.3	8.15	NS
4692	Neuraminidase (sialidase)	11.7	23.7	2.98	0.016
4677	Pectin methylesterase	54.3	63.7	7.93	NS
4806	Predicted neuraminidase (sialidase) L-rhamnose isomerase	26	50.7	6.77	0.022
5026	Hexokinase	7.3	8.3	2.36	NS

* *Cellulase. NS: Not Significant*.

**Table 8:** **Lipid metabolism and transport clusters of orthologous genes (COG) categories showing significant read abundance differences within the primary (1 h) and secondary (4 h) bacteria attached to perennial ryegrass**.

**COG number**	**Function**	**Average COG normalized read abundance**			
		**1 h**	**4 h**	**SED**	***P***
20	Undecaprenyl pyrophosphate synthase	34.7	71.7	13.6	0.053
183	Acetyl-CoA acetyltransferase	45.7	89.3	17.95	NS
204	1-acyl-sn-glycerol-3-phosphate acyltransferase	31	42.3	7.54	NS
245	2C-methyl-D-erythritol 2,4-cyclodiphosphate synthase	38	79.7	14	0.041
331	(Acyl-carrier-protein) S-malonyltransferase	37	69	11.8	0.053
332	3-Oxoacyl-[acyl-carrier-protein] synthase III	57.3	91.7	10.3	0.029
365	Acyl-coenzyme A synthetases/AMP-(fatty) acid ligases	106	211	28.1	0.02
416	Fatty acid/phospholipid biosynthesis enzyme	19.7	47.7	9.5	0.043
439	Biotin carboxylase	47	96.7	14.3	0.026
511	Biotin carboxyl carrier protein	36	107.3	14.7	0.008
558	Phosphatidylglycerophosphate synthase	11.7	27.7	5.7	0.048
575	CDP-diglyceride synthetase	67	136.3	16	0.012
615	Cytidylyltransferase	26.7	45.7	7.76	NS
623	Enoyl-[acyl-carrier-protein] reductase (NADH)	5.33	4.67	1.886	NS
657	Esterase/lipase	75	142	21.7	0.037
671	Membrane-associated phospholipid phosphatase	19.7	31	6.84	NS
688	Phosphatidylserine decarboxylase	13.3	24.7	8.08	NS
764	3-Hydroxymyristoyl/3-hydroxydecanoyl-(acyl carrier protein) dehydratases	22	39	11.63	NS
777	Acetyl-CoA carboxylase beta subunit	13	28.7	8.19	NS
821	Enzyme involved in the deoxyxylulose pathway of isoprenoid biosynthesis	61	146	25.3	0.028
825	Acetyl-CoA carboxylase alpha subunit	16	22.3	9.63	NS
1022	Long-chain acyl-CoA synthetases (AMP-forming)	92.3	133.3	3.7	<0.001
1024	Enoyl-CoA hydratase/carnithine racemase	25.7	45.3	8.48	NS
1154	Deoxyxylulose-5-phosphate synthase	109	219	40.1	0.051
1182	Acyl carrier protein phosphodiesterase	6	6.67	1.202	NS
1183	Phosphatidylserine synthase	8.3	13.3	3.5	NS
1211	4-Diphosphocytidyl-2-methyl-D-erithritol synthase	77	175	20.8	0.009
1250	3-Hydroxyacyl-CoA dehydrogenase	21.7	47	6.5	0.018
1257	Hydroxymethylglutaryl-CoA reductase	4.3	13.7	2.5	0.02
1260	Myo-inositol-1-phosphate synthase	27	50	5.2	0.011
1502	Phosphatidylserine/phosphatidylglycerophosphate/cardiolipin synthases and related enzymes	62.7	95.7	10.8	0.038
1562	Phytoene/squalene synthetase	4	4.33	1.453	NS
1577	Mevalonate kinase	3	10.3	2.4	0.038
1607	Acyl-CoA hydrolase	4	4.7	2.03	NS
1657	Squalene cyclase	3.67	6	1.453	NS
1788	Acyl CoA:acetate/3-ketoacid CoA transferase, alpha subunit	3.33	2	1.667	NS
1835	Predicted acyltransferases	9	17.3	4.01	NS
1884	Methylmalonyl-CoA mutase, N-terminal domain/subunit	132	196	21.7	0.042
1924	Activator of 2-hydroxyglutaryl-CoA dehydratase (HSP70-class ATPase domain)	155	277	57.5	NS
1946	Acyl-CoA thioesterase	1.7	10.7	9.18	NS
1960	Acyl-CoA dehydrogenases	45	92	15.1	0.036
2030	Acyl dehydratase	7	12.7	2.33	NS
2031	Short chain fatty acids transporter	2	7	0.6	<0.001
2057	Acyl CoA:acetate/3-ketoacid CoA transferase, beta subunit	3	11	3.11	NS
2067	Long-chain fatty acid transport protein	9.3	14	2.73	NS
2084	3-Hydroxyisobutyrate dehydrogenase and related beta-hydroxyacid dehydrogenases	22	36.3	6.94	NS
2185	Methylmalonyl-CoA mutase, C-terminal domain/subunit (cobalamin-binding)	123.3	184.3	16.5	0.021
2267	Lysophospholipase	30	40	6.53	NS
2272	Carboxylesterase type B	107	192	21.9	0.018
3000	Sterol desaturase	8.7	5	2.4	NS
4799	Acetyl-CoA carboxylase, carboxyltransferase component (subunits alpha and beta)	63	102	14.3	0.053
4981	Enoyl reductase domain of yeast-type FAS1	2.33	1.33	0.745	NS

NS: Not Significant.

**Table 9:** **Perennial ryegrass attached rumen bacterial functional Kyto encyclopedia of genes and genomes (KEGG) pathways present following either 1 or 4 h of rumen incubation only, and in relation to boxed areas shown in Figure 5**.

**Box no**	**Present (h)**	**EC number**	**Classification**	**Pathway**
1	1	2.3.1.68	Glutamine N-acyltransferase	Biosynthesis of secondary metabolites
1	1	2.1.1.68	Caffeate methyltransferase	Biosynthesis of secondary metabolites
1	4	1.14.19.3	Delta6-desaturase	Linoleic acid metabolism/biosynthesis of unsaturated fatty acids
2	4	1.14.14.1	Cytrochrome P450	Arachidonic acid metabolism
2	1	3.10.1.1	N-sulfoglucosamine sulfohydrolase	Glucosaminoglycan degradation/lysozyme
2	1	2.4.1.155	Alpha-1,3(6)-mannosylglycoprotein	N-glycan biosynthesis
2	1	3.1.6.12	Arylsulfatase	Glycosaminoglycan degradation/lysosome
2	1	3.1.1.23	Acylglycerol lipase	Glycerolipid metabolism/retrograde endocannabinoid signaling
2	1	1.1.1.101	Acylglycerone-phosphate reductase	Glycerophospholipid metabolism/Ether lipid metabolism
2	4	2.7.8.2	Diacylglycerol cholinephosphotransferase	Phosphonate and phosphinate/glycerophospholipid/ether lipid metabolism
2	4	2.1.1.17	Phosphotidylethanolamine N-methyltransferase	Glycerophospholipid metabolism/synthesis of secondary metabolites
2	4	2.7.8.20	Glycerophosphotransferase	Glycerolipid metabolism
2	4	3.1.3.66	Inositol polyphosphate-4-phosphatase	Inositol phosphate metabolism/phosphatidylinositol signaling system
3	1	3.2.1.20/3.2.1.3	Maltose glucoamylase	Galactose/starch and sucrose metabolism/carbohydrate digestion and absorption
3	1	2.4.1.17	Glucuronosyltransferase	Pentose and glucoronate interconversions/ascorbate metabolism/steroid synthesis etc.
3	1	1.1.1.43	Phosphogluconate 2-dehydrogenase	Pentose/glutathione phosphate pathway/microbial metabolism in diverse environments
3	4	4.1.2.29	6-Phospho-5-dehydro-2 deoxy-D gluconate aldolase	Inositol phosphate metabolism
3	4	2.7.1.60	N-acyl mannosamine kinase	Amino sugar and nucleotide sugar metabolism
3	4	2.7.1.13	Dehydrogluconokinase	Pentose phosphate pathway
3	4	4.4.1.16	Selenocysteine lyase	Selenocompound metabolism
3	4	1.1.1.87	Homoisocitrate dehydrogenase	Lysine biosynthesis/microbial metabolism in diverse environments/biosynthesis antibiotics
4	1	1.14.13.178	Oxygen oxidoreductase	Biosynthesis of secondary metabolites/Microbial metabolism in diverse environments
4	1	2.3.1.5	Acrylamine N-acetyltransferase	Nitrotoluene degradation/Drug metabolism
4	1	3.5.3.4	Allantoicase	Purine metabolism/microbial metabolism diverse environments
4	4	1.14.18.1	Tyrosinase	Tyrosine/riboflavin metabolism/Biosynthesis of secondary metabolites
4	4	1.10.3.1	Catechol oxidase	Tyrosine metabolism/Biosynthesis of secondary metabolites
4	4	1.14.16.2	Tyrosine 3-monooxygenase	Tyrosine metabolism
4	4	1.4.3.4	Monochrome oxidase	Amino acid metabolism/Biosynthesis of secondary metabolites
4	4	3.7.1.5	Acylpyruvate hydrolase	Tyrosine metabolism/Microbial metabolism in diverse environments
4	4	3.7.1.3	Kynureninase	Tryptophan metabolism
4	4	4.1.1.43	Phenylpyruvate decarboxylase	Phenylalanine and Tryptophan metabolism
5	1	5.4.99.77	Inosterol synthase	Steroid biosynthesis/Biosynthesis of secondary metabolites
5	1	1.1.1.62/1.1.1.239	Beta-estradiol A-dehydrogenase	Steroid hormone biosynthesis
5	1	1.14.13.50	Pentachlorophenol monooxygenase	Chlorocyclohexane, chlorobenzene, fluorobenzoate degradation/Microbial metabolism in diverse environments
5	1	1.1.1.46	Glutathionine-independent formaldehyde dehydrogenase	Chloroalkane and chloroalkene degradation/Methane metabolism
5	1	1.13.11.2	Catechol 2,3 dioxygenase	Chlorocyclohexane, chlorobenzene, benzoate, xylene, styrene degradation/Microbial metabolism in diverse environments
5	1	1.13.11.39	Biphenyl-2,3-diol 1,2 dioxygenase	Chlorocyclohexene, chlorobenzene, dioxin degradation/Degradation aromatic compounds
5	1	1.2.1.32	Aminomuconate-semialdehyde dehydrogenase	Tryptophan metabolism
5	1	1.13.11.37	Hydroxyphenol 1,2 diooxygenase	Chlorohexane, chlorobenzene, benzoate degradation/Microbial metabolism in diverse environments
5	1	4.1.1.55	4,5-Dihydroxyphthalate decarboxylase	Polycyclic aromatic hydrocarbon degradation/ Microbial metabolism in diverse environments
5	4	1.3.1.3	3-Oxo-5-beta-sterol 4-dehydrogenase	Steroid hormone biosynthesis
5	4	5.5.1.1	Muconate cycloisomerase	Chlorocyclohexane, chlorobenzene, benzoate, fluorobenzoate, toluene degradation/degradation of aromatic compounds
5	4	1.14.13.1	Salicylate 1-monooxygenase	Dioxine, polycyclic aromatic hydrocarbon, naphthalene degradation/Microbial metabolism in diverse environments
5	4	5.5.1.2	3-Carboxy-cis, cis-muconate cycloisomerase	Benzoate degradation/Degradation of aromatic compounds
5	4	3.1.1.24	3-Oxoadipate enol-lactonase	Benzoate degradation/Degradation of aromatic compounds
5	4	1.2.1.7	Benzaldehyde dehydrogenase	Xylene, toluene, aminobenzoate degradation/Microbial metabolism in diverse environments
5	4	3.5.99.4	N-isopropylammedide isopropyl aminohydrolase	Atrazine degradation
5	4	1.14.13.20	2,4-Dichlorophenol 6-monooxygenase	Chlorocyclohexene and chlorobenzene degradation/Microbial metabolism in diverse Environments
5	4	3.5.1.54	Allophenate hydrolase	Arginine biosynthesis/ Atrazine degradation/ Microbial metabolism in diverse environments
6	1	1.13.11.2	Catechol 2,3 dioxygenase	Degradation of aromatic compounds/Microbial metabolism in diverse environments
6	1	4.1.1.47	Tartamate-semialdehyde synthase	Glyoxylate and dicarboxylate metabolism
6	1	1.2.1.31	L-amino adipate-semialdehyde dehydrogenase	Lysine biosynthesis and degradation/ Biosynthesis of amino acids
6	4	4.1.1.8	Oxalyl-CoA decarboxylase	Glyoxylate and dicarboxylate metabolism
6	4	2.6.1.44/2.6.1.40	Alanine-glyoxylate transaminase	Amino acid metabolism and degradation
6	4	5.1.99.1	Methylmalonyl-CoA	Valine, leucine, isoleucine degradation/Propanoate metabolism/ Carbon metabolism
6	4	1.3.1.32	Maleylactetate reductase	Chlorocyclohexane, chlorobenzene, benzoate, fluorobenzoate, and toluene degradation/microbial metabolism in diverse environments
7	1	2.6.1.11/2.6.1.17	Acetylornithine/N-Succinyldiaminopimelate aminotransferase	Biosynthesis of amino acids/ 2-oxocarboxylic acid metabolism
7	1	1.3.99.12	Short/branched chain acyl-CoA dehydrogenase	Valine, leucine, and isoleucine degradation
7	1	2.3.1.178	L-2,4-diaminobutyric acid acetyl transferase	Glycine, serine, and threonine metabolism
7	1	3.5.3.4	Allentoicase	Purine metabolism/Microbial metabolism in diverse environments
7	1	6.3.4.16	Carbamoyl-phosphate synthase (ammonia)	Biosynthesis amino acids/Nitrogen and carbon metabolism
7	1	6.3.2.5	Phosphopantothinate cysteine ligase	Pantotholate and CoA biosynthesis
7	4	4.2.1.33	3-Isopropylmalate dehydratase	Amino acid biosynthesis/ 2-oxocarboxylic acid metabolism
7	4	3.5.1.54	Allophonate hydrolase	Arginine biosynthesis/Atrazine degradation/Microbial metabolism in diverse environments
7	4	1.4.3.4	Monoamine oxidase	Amino acid metabolism/Isoquinolone alkaloid biosynthesis/Biosynthesis of secondary metabolites

### Plant chemical changes

**Figure 5:**
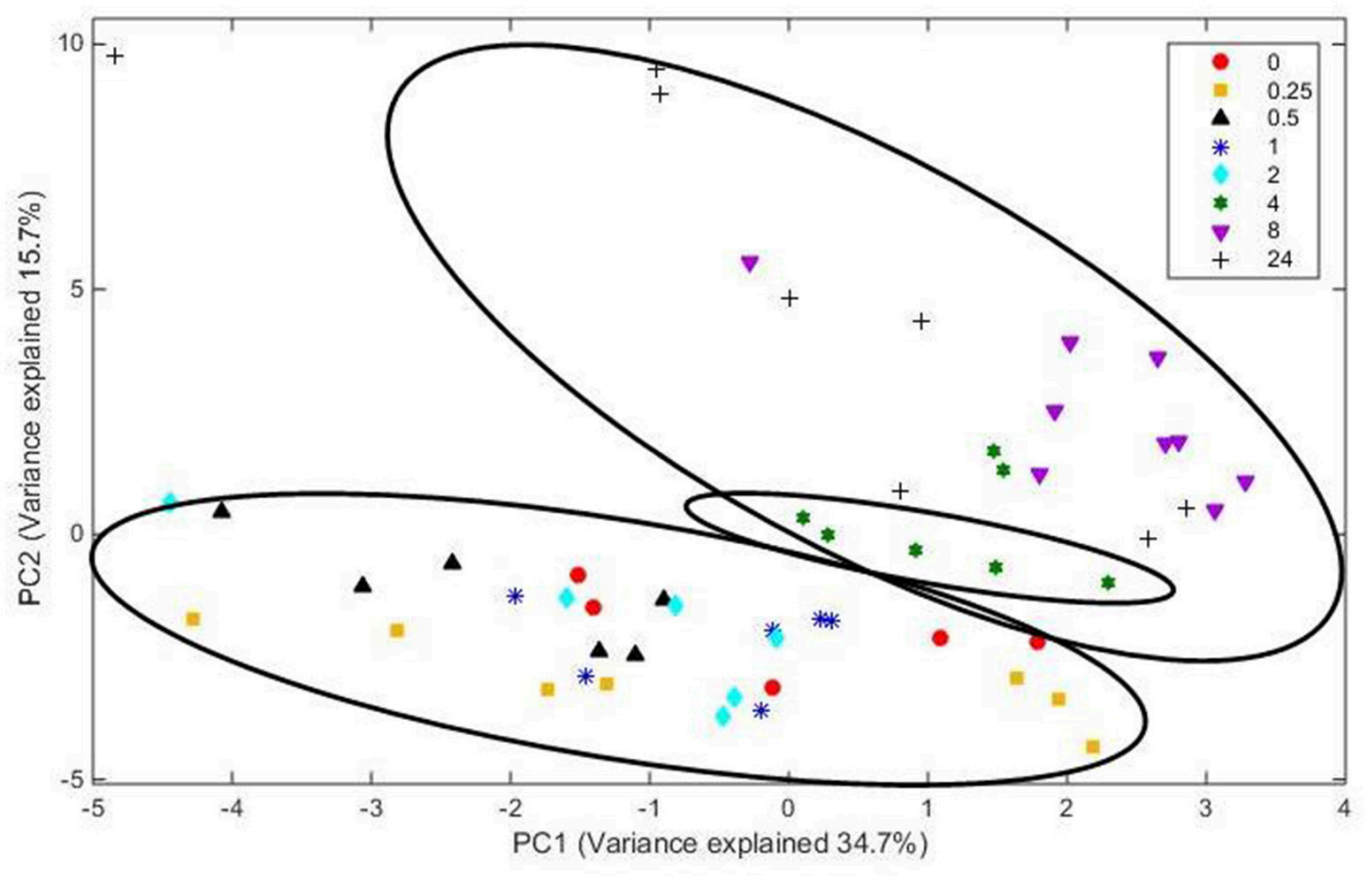
**Score plot of principal components PC 1 vs. PC 2 for plant material from which the attached microbes had been removed**. Score data sets are of 60 spectra from three analytical replicates and at least two spectral analyses. Circles indicate clusters.

**Figure 6:**
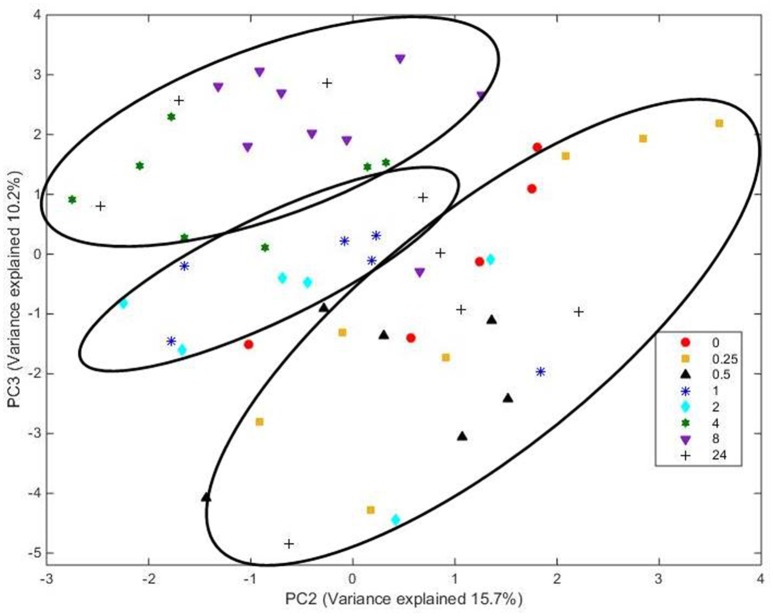
**Score plot of principal components PC 2 vs. PC 3 for plant material from which the attached microbes had been removed**. Score data sets are of 60 spectra from three analytical replicates and at least two spectral analyses. Circles indicate clusters.

Analysis of FT-IR spectra of residual plant material taken over time, showed that the scores for the first 20 principal components (together accounting for 95% of variance) were significantly different between spectra from different time points (*P* < 0.001), with plots of the scores for PC1 vs. PC2 (Figure 5) and PC2 vs. PC3 (Figure 6) showing clear clustering between spectra from samples incubated for up to 2 h compared with those incubated for longer periods. It should be noted that the circles drawn to denote clustering have been constructed by eye for ease of interpretation, and not using any statistical methodology. PC 1 accounted for 34.7% of total variance, PC2 accounted for 15.7%, and PC3 10.2%. The spectra for all samples at various time points were similar (Supplementary Figure 3). Analysis of the loadings for PC 1 showed positive contributions (i.e., a decrease in chemical content) from variables at 950 and 1035 cm^−1^; the first one of which has been reported to be associated with cellulose or possibly galactan (Kacurakova et al., 2000; Alonso-Simon et al., 2004; Supplementary Figure 3), and the second to pectin or xyloglucan (Kacurakova et al., 2000; Alonso-Simon et al., 2004; Supplementary Figure 3). Negative contributions (i.e., and increase in chemical content) to PC1 were present at 1393 and 1598 cm^−1^, possibly being attributable to the amino amide I absorption of proteins (Schmitt and Flemming, 1998; Supplementary Figure 3). The loadings of PC2 showed similarities with PC1 but additional positive contributions were detected at 1029 cm^−1^, a region that has also been associated with cellulose content (Kacurakova et al., 2000; Alonso-Simon et al., 2004; Supplementary Figure 3) and 1664 cm^−1^, which is in the amide I absorption region of amino groups. PC2 was negatively correlated with absorptions at 1394 and 1591 cm^−1^ (Supplementary Figure 3), PC 2 was positively correlated with absorbances at 2908 and 2850 cm^−1^, which have been associated with the asymmetric and symmetric stretching on CH_2_ moieties in fatty acids (Schmitt and Flemming, 1998; Supplementary Figure 3). The loadings for PC3 show positive contributions at 1359 cm^−1^, corresponding to a CH2 stretch of cellulose (Kacurakova et al., 2000), and several peaks relating to hemicellulose components and pectin at 979, 995, 1045, and 1080 cm^−1^ (Supplementary Figure 3). PC3 was also negatively correlated with peaks at 161 and 1529 cm^−1^, corresponding to amide 1 and amide 11 in proteins (Schmitt and Flemming, 1998; Supplementary Figure 3).

## Discussion

In this study, we demonstrate that colonization of PRG by rumen bacteria is biphasic with primary (up to 4 h) and secondary (post 4 h) events observed based on changes seen in attached bacterial diversity. We also demonstrate that these changes in diversity correlate with changes in functional capacity and changes in the metabolome of PRG itself. Thus, despite the resilience and redundancy observed within the rumen microbiome it is apparent, on a DNA level, that diversity and function are linked.

In terms of temporal diversity of the bacteria attached to PRG, the phyla Firmicutes and Bacteroidetes dominated and changes in phyla level diversity were not evident over time. In our previous study investigating changes in diversity of bacteria attached to PRG (16S rRNA based) over time within the rumen, we also noted that Firmicutes and Bacteroidetes dominated, but Fibrobacteres sequence abundances were higher than noted in this study (4% compared with an average of 0.6% in this study; Huws et al., 2016). Piao et al. (2014) when investigating diversity (16S rDNA sequencing) of rumen bacteria attached to switchgrass also noted dominance of Firmicutes and Bacteroidetes irrespective of time. On an order level we found that the Clostridiales, Selemonadales, and Bacteroidales were the most abundant, and changes in order level diversity were not evident over time. Previously we also noted that Clostridiales, Selemonadales, and Bacteroidales were dominant, but also reported that 16S rRNA read abundances of Fibrobacterales, Coriobacterales, and Spirochaetales were also reasonably abundant, representing 4, 3, and 2% of the total sequences reads, respectively (Huws et al., 2016). In this study, read abundances for Fibrobacterales, Coriobacterales, and Spirochaetales were represented 0.6, 1.5, and 1.5% of total reads, respectively. The study conducted by Piao et al. (2014) on the bacteria attached to switchgrass over time also showed similar results to those in our study. On a family level we found that the most abundant classified families were *Lachnospiraceae, Veillonellaceae, Prevotellaceae, Eubacteriaceae, Clostridiaceae*, and significant (*P* > 0.05) increases in *Bacillaceae, Lachnospiraceae, Porphyromonadaceae*, and *Prevotellaceae* were seen during secondary colonization events compared with abundances present during primary colonization. Previously we also noted that *Lachnospiraceae, Veillonellaceae, Prevotellaceae*, and *Ruminococcaceae* were dominant, but we also reported that 16S rRNA read abundances of *Fibrobacteraceae* and *Coriobacteriaceae* were reasonably abundant, representing 5 and 2% of the total sequence reads, respectively (Huws et al., 2016). In this study read abundances for *Fibrobacteraceae*, and *Coriobacteriaceae* represented 0.6 and 1.5% of total reads, respectively. Thus, *Coriobacteriaceae* read abundances were reasonably similar between both studies but *Fibrobacteraceae* were lower in this study. We also found that read abundance for *Eubacteriaceae* and *Clostridiaceae* were higher in this study than our previous study (Huws et al., 2016). Again, the study conducted by Piao et al. (2014) on bacteria attached to switchgrass showed similar results to this study in terms of the most abundant families. In this study, we saw increases in *Bacillaceae, Lachnospiraceae, Porphyromonadaceae*, and *Prevotellaceae* during secondary colonization compared with abundances present during primary colonization. We noted increases in *Lachnospiraceae* only between primary and secondary colonization events in our previous study (Huws et al., 2016). On a genus level we found that the most abundant classified genera were *Butyrivibrio, Selenomonas, Prevotella, Eubacterium, Pseudobutyrivibrio*, and *Ruminococcus*, with significant (*P* > 0.05) increases in *Acidaminococcus, Bacillus, Blautia, Butyrivibrio*, and *Prevotella* seen during secondary colonization compared with abundances present during primary colonization. In our previous study we also noted that *Butyrivibrio, Selenomonas, Prevotella, Pseudobutyrivibrio*, dominated the attached microbiota irrespective of time (Huws et al., 2016). We also found that *Olsenella* and *Fibrobacter* were reasonably dominant previously, which was not apparent in this study. The previous study also indicated that *Pseudobutyrivibrio* increased in abundance during the secondary phase of colonization. Again, the study conducted by Piao et al. (2014) showed similar results to those in this study in terms of the most abundant bacterial genera attached to switchgrass over time. The similarities between our *in vitro* study and these *in sacco* studies (Piao et al., 2014; Huws et al., 2016) demonstrate that *in vitro* rumen incubations are reasonably representative of attachment events that occur within the rumen itself. Also, the use of shotgun metagenomic sequencing in these studies, as compared with results from other studies using 16S rRNA (RNA and DNA) based sequencing illustrates that non-amplification based techniques are beneficial for taxonomical identification as well as allowing insight into the functionality of the rumen microbiota.

In terms of the temporal functional capacity of the attached bacteria, there was a clear difference between the function of the primary attached bacteria and that of the secondary attached bacteria. The main functions seen were broadly within the COG categories metabolism, information, storage and processing, and cellular processes and signaling with gene abundances in all three of these categories being higher at 4 h of colonization compared with abundances at 1 h of colonization. Specifically, amino acid, carbohydrate, and lipid storage and transport were the main functionalities demonstrated by the attached bacteria (all residing within the function metabolism). Most of the genes within amino acid, carbohydrate and lipid storage and transport functional categories were increased in abundance during secondary colonization. KEGG pathway analysis showed that most pathways were present within PRG attached bacteria following both 1 and 4 h of rumen incubation, thus this coupled with the COG abundance data suggests that secondary colonization events is associated largely with increases in abundance of genes present during primary colonization. These increases correlate with increases in the genera *Acidaminococcus, Bacillus, Butyrivibrio*, and *Prevotella* suggesting that these are the bacteria responsible for the increase in amino acid, carbohydrate and lipid metabolism seen during secondary colonization. *Acidaminococcus* are asaccharolytic but have the capacity of producing ammonia (Eschenlauer et al., 2002). *Butyrivibrio* spp. are also known for their proteolytic, biohydrogenating, and carbohydrate degradation within the rumen context (Hobson and Stewart, 1997; Krause et al., 2003). Rumen *Prevotella* spp. are often referred to as amylolytic and proteolytic, but they also have carbohydrate metabolic capacity (Gardener et al., 1995; Krause et al., 2003; Accetto and Avguštin, 2015; Kishi et al., 2015). Interestingly, the number of COG carbohydrate families classifying as cellulases was on average only 3.2% of the total normalized reads within those sequences classified as COG families involved in carbohydrate metabolism. Conversely in the study by Hess et al. (2011), 23% of the glycosyl hydrolases identified in the switchgrass attached bacteria post 24 h of rumen incubation were putative cellulases. The sequencing depth in the study by Hess et al. (2011) was 268 GB whereas we obtained on average 0.9 GB which may explain the differential in identifiable cellulases, coupled with the use of different forages substrates. Nonetheless, it is more likely a consequence of the fact that Hess et al. (2011) harvested bacteria from 24 h incubations when fermentation will be at a very advanced state compared to our study. Indeed, DM degradation in this study show that by 24 h 76% of the plant material was degraded as compared to 35% at 8 h of incubation. Irrespective, our data is suggestive that changing the cell wall characteristics of the plant material to focus on decreasing recalcitrance of structural carbohydrates may allow more efficient breakdown of the cell wall and increase speed of bioavailability of intra-plant nutrients to the microbes and the ruminant. Conversely, developing novel strategies to increase the cellulases, particularly endocellulase, capacity of the microbiota may result in more efficient breakdown of the cell wall and increases speed of bioavailability of intra-plant nutrients to the microbes and the ruminant.

Multivariate analysis of the FT-IR spectra corroborated the metagenomic data by showing increases in carbohydrates, amino acids, and lipid metabolism from primary to secondary colonization. The plant protein and lipid changes may have occurred within the plant itself irrespective of having an attached microbial community as we know that fresh forage is capable of degrading its own protein and lipids within the first 2 h of ruminal incubation (Kingston-Smith et al., 2003, 2008; Lee et al., 2004). A recent publication by Kingston-Smith et al. (2013) used FT-IR to investigate the metabolite fingerprint of the interactome generated during colonization of fresh PRG. In that work, richness of the spectra derived from a combination of metabolic activities of plant and bacterial chemistries (forage plus attached bacteria) meant that analysis of the resultant metabolite profiles did not demonstrate clear differences between 2 and 4 h although a slight change from 8 h onwards was noted. Hence, the results reported here further our understanding of forage degradation by illustrating the changes in plant chemistry that are specifically associated with sequential microbial colonization events when fresh forage is incubated under rumen-like conditions.

In conclusion, the data obtained in this study illustrate that temporal changes in the diversity of bacteria attached to PRG, between primary (up to 4 h) and secondary (post 4 h) colonization events, correlate with increases in amino acid, carbohydrate and lipid storage and transport functional capacity. This data suggests that these changes in gene abundance result in increased metabolism of plant amino acids, carbohydrates and lipids during secondary colonization events. The data suggests that the capacity of the rumen microbes to degrade the more recalcitrant components of the plant cell wall may be the rate limiting factor in increasing bioavailability of nutrients to the microbes and ultimately the ruminant during the first 4 h post-ingestion. Future strategies to increase ruminant nutrient use efficient should investigate the benefits of reducing the recalcitrant nature of the plant cell wall and/or increasing the cellulolytic capacity of the rumen microbiome within early colonization events in particular.

## Author contributions

SH, EK, AK, MT, and CN conceived the project. OM completed the laboratory work under supervision of SH and EK. TW, MH and SH completed the sequencing and downstream analysis of the sequences. SH wrote the paper with input from all co-authors. GA helped OM with FT-IR analysis.

## Funding

We acknowledge funding from COLCIENCIAS, CORPOICA (Colombia) and the Biological Sciences Research Council, UK via grant number BB/J0013/1; BBS/E/W/10964A-01. SH is also funded 75% by the Coleg Cymraeg Cenedlaethol.

### Conflict of interest statement

The authors declare that the research was conducted in the absence of any commercial or financial relationships that could be construed as a potential conflict of interest.

## Supplementary material

Supplementary Table 1 **Average sequence summary information obtained from attached primary bacterial biofilm communities over all time points**.

Supplementary Figure 1 **Representative PCR-DGGE derived un-weight pair group method with arithmetic mean (UPGMA) dendograms showing temporal attached bacterial diversity in the presence of fresh perennial ryegrass for experimental replicate 1 (Replicate 2 and 3 showed very similar results)**. The numbers represent the different incubation times and scale relates to percent similarity.

Supplementary Figure 2 **Rarefaction curve showing metagenomic sequencing depth obtained for each sample**.

Supplementary Figure 3 **FT-IR normalized spectra showing the change in signal intensity (absorbance) for decolonized plant material (plant material with the attached microbes removed) as function of incubation time**. Spectral data are from 60 spectra from three analytical replicates and at least two spectral analyses.
