# Supplementary material for: Temporal Metagenomic and Metabolomic Characterization of Fresh Perennial Ryegrass Degradation by Rumen Bacteria
Source: Front Microbiol. 2016 Nov 18;7:1854. doi: 10.3389/fmicb.2016.01854 (PMC5114307; doi:10.3389/fmicb.2016.01854)
Supplement: Supplementary Table 1 — Average sequence summary information obtained from attached primary bacterial biofilm communities over all time points. [file Table1.DOCX]

**Supplementary Table 1** Average sequence summary information obtained from attached primary bacterial biofilm communities over all time points.

| **Sequence information** | **Average (SED)** |
| --- | --- |
| Pre QC: Base pair (bp) Count | 1,167,170,697 (294,618,469) |
| Pre QC: Sequences Count | 7,309,079 (1,887,929) |
| Pre QC: Mean Sequence Length | 160 (2) |
| Pre QC: Mean GC percent | 44 (1) |
| Artificial Duplicate Reads: Sequence Count | 1,694,151 (612,528) |
| Post QC: bp Count | 903,566,009 (294,384,197) |
| Post QC: Sequences Count | 5,543,494 (1,283,997) |
| Post QC: Mean Sequence Length | 163 (2) |
| Post QC: Mean GC percent | 45 (0) |
| Processed: Predicted Protein Features | 3,001,473 (623,043) |
| Processed: Predicted rRNA Features | 304,081 (51,604) |
| Alignment: Identified Protein Features | 516,365 (140, 981) |
| Alignment: Identified rRNA Features | 2,915 (55) |
| Annotation: Identified Functional Categories | 207,034 (70,889) |

SED: Standard deviation
