# Supplementary figures and images for: Temporal Metagenomic and Metabolomic Characterization of Fresh Perennial Ryegrass Degradation by Rumen Bacteria
Source: Front Microbiol. 2016 Nov 18;7:1854. doi: 10.3389/fmicb.2016.01854 (PMC5114307; doi:10.3389/fmicb.2016.01854)

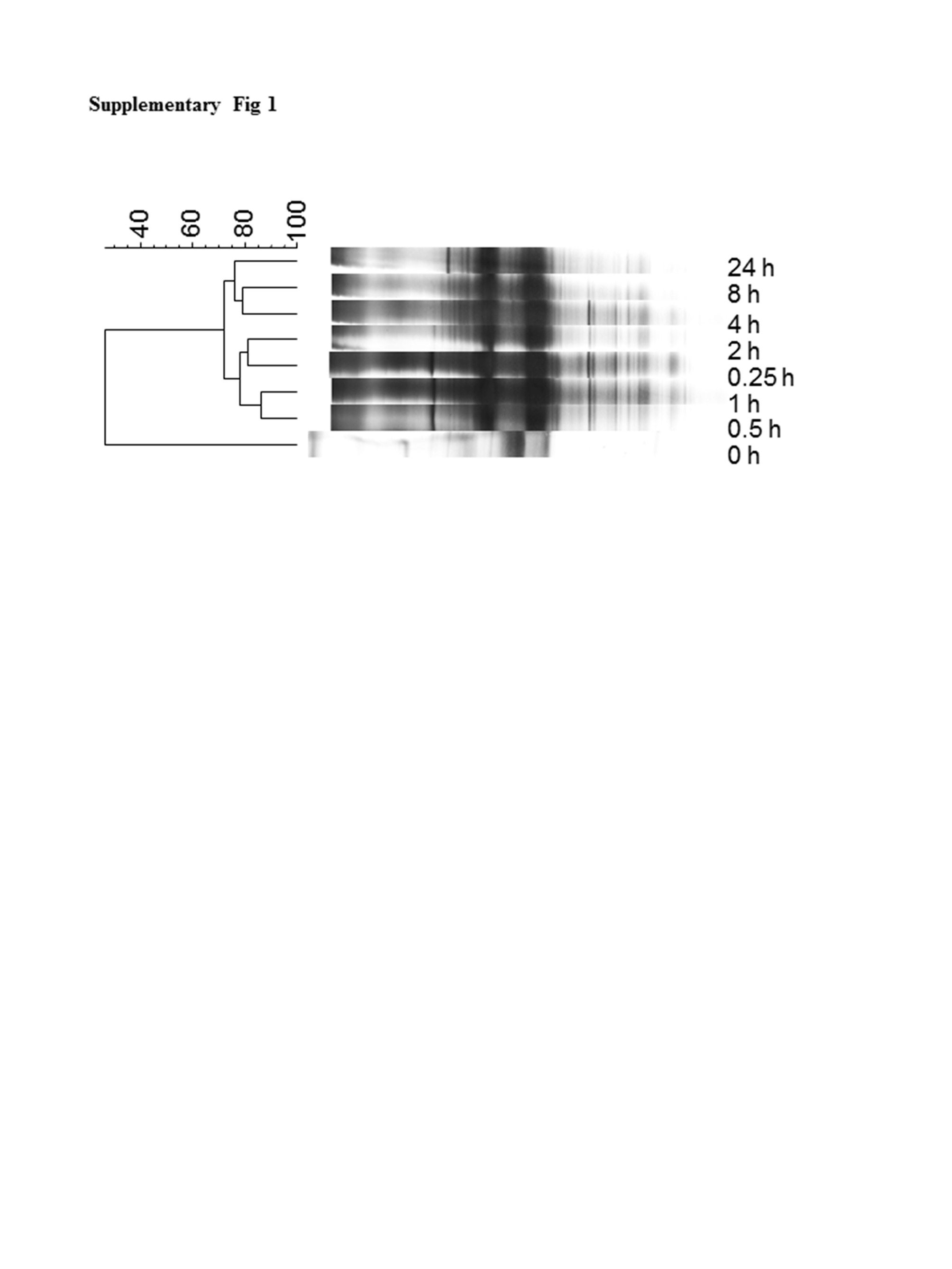

Supplement: Supplementary Figure 1 — Representative PCR-DGGE derived un-weight pair group method with arithmetic mean (UPGMA) dendograms showing temporal attached bacterial diversity in the presence of fresh perennial ryegrass for experimental replicate 1 (Replicate 2 and 3 showed very similar results). The numbers represent the different incubation times and scale relates to percent similarity. [file Image1.TIF]

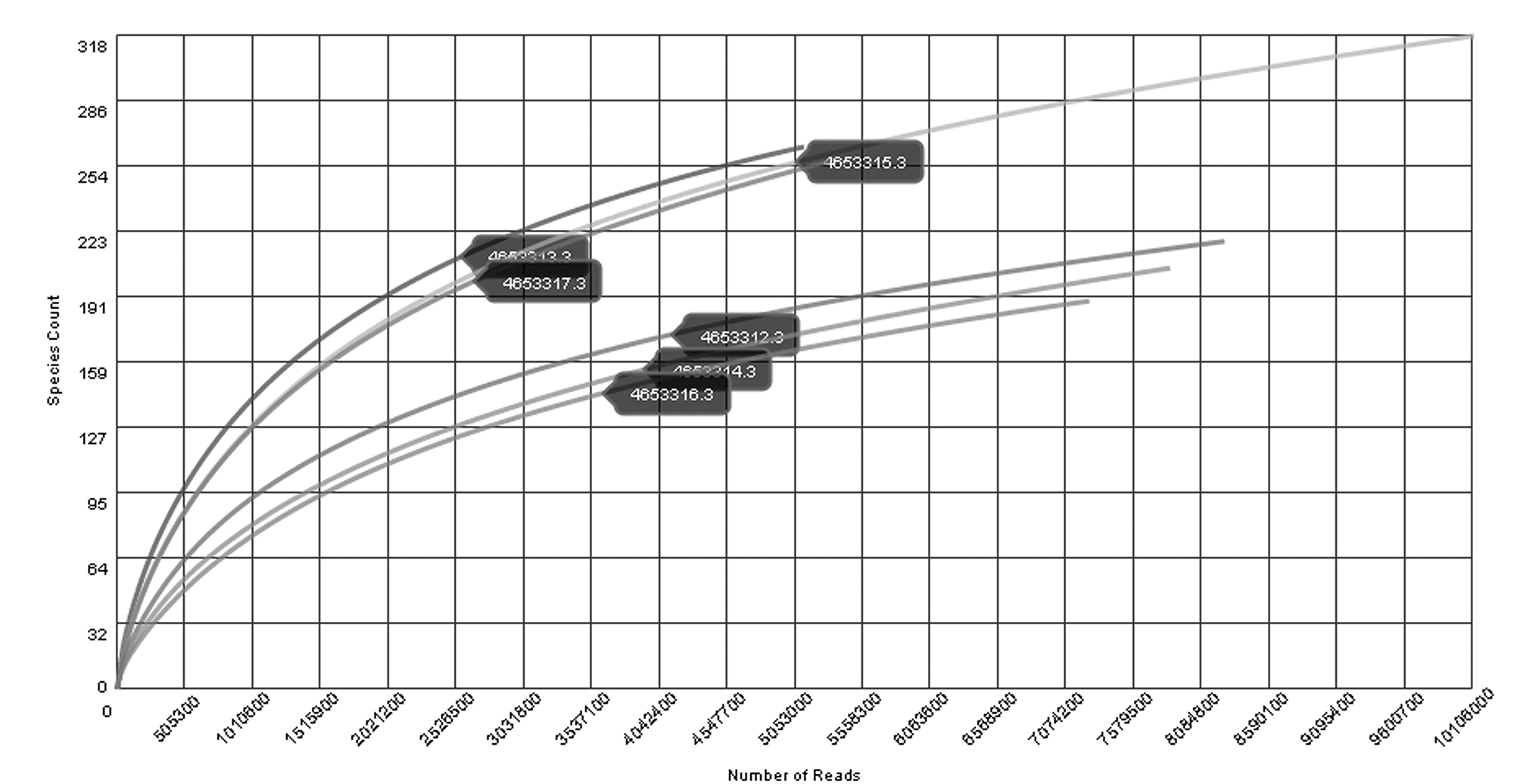

Supplement: Supplementary Figure 2 — Rarefaction curve showing metagenomic sequencing depth obtained for each sample. [file Image2.TIF]

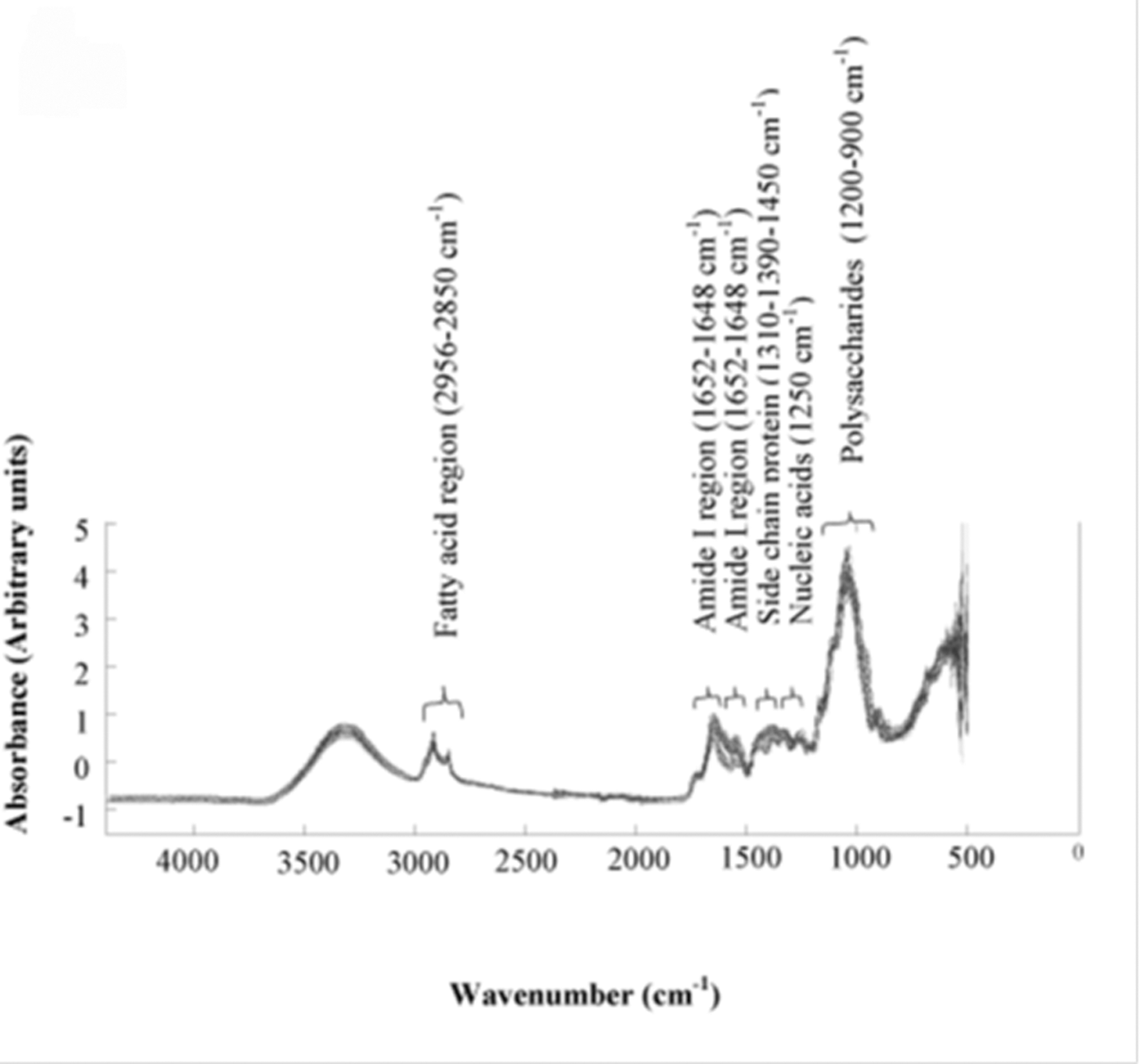

Supplement: Supplementary Figure 3 — FT-IR normalized spectra showing the change in signal intensity (absorbance) for decolonized plant material (plant material with the attached microbes removed) as function of incubation time. Spectral data are from 60 spectra from three analytical replicates and at least two spectral analyses. [file Image3.TIF]
